# The phylogeography of Middle Eastern tree frogs in Israel

**DOI:** 10.1038/s41598-024-52700-5

**Published:** 2024-02-02

**Authors:** Gal Mesika Surizon, Eli Geffen, Uri Roll, Sarig Gafny, R. G. Bina Perl

**Affiliations:** 1 Faculty of Marine Sciences, Ruppin Academic Center, 40297 Mikhmoret, Israel; 2 School of Zoology, Tel Aviv University, 69978 Tel Aviv, Israel; 3 Mitrani Department of Desert Ecology, The Jacob Blaustein Institutes of Desert Research, Ben-Gurion University of the Negev, 8499000 Midreshet Ben-Gurion, Israel; 4 Department of Terrestrial Zoology, Senckenberg Research Institute and Natural History Museum Frankfurt, Senckenberganlage 25, 60325 Frankfurt, Germany

**Keywords:** Genetics, Zoology

## Abstract

Western Palearctic treefrogs of the genus *Hyla* provide an example of a morphologically and ecologically cryptic group. Up to three distinct *Hyla* species have been proposed as resident in Israel and this number has consistently been subject to taxonomical debates. Here, we analyzed 16S rRNA and COI gene fragments of 658 individuals sampled at 47 pools in nine regions across Israel and the West Bank in order to resolve the taxonomic status of *Hyla* frogs. We generated both Bayesian and Maximum Likelihood phylogenies, and constructed time-calibrated trees to provide an evolutionary and historical context of sequence variations. We further applied SAMOVA as well as Monmonier’s maximum-difference algorithm to study the genetic structure among populations and to identify potential zones acting as barriers to gene flow across locations. Our results revealed two distinct haplogroups for each gene fragment, with 95% CI divergence times dated from 8.9–17.1 Mya (16S) and 7.1–23.6 Mya (COI), respectively. SAMOVA and barrier analyses partitioned the populations into three groups. Our results highlight that, while there are probably only two *Hyla* species in Israel, one population of one of the species might qualify as a separate evolutionarily significant unit. Our findings elucidate the taxonomic status of *Hyla* frogs in Israel and provide the basis for determining appropriate management and conservation priorities.

## Introduction

Assessing and inventorying biodiversity is a prerequisite for establishing appropriate management and conservation policies. Only after the taxonomic status of populations has been clarified and reliable information on distribution ranges is available, can the acquired knowledge be used to efficiently plan and apply conservation actions. However, there may not be clear morphological differences between otherwise genetically distinct species. Consequently, taxonomy in regard to cryptic species complexes often remains a source of confusion and controversy until it can be resolved on the basis of molecular data. Cryptic species are found in all classes of vertebrates^1–5^, and are especially common in amphibians, which, unlike many other vertebrates, have a relatively conservative body plan and thus a small number of external characters conducive to species identification^6^.

Despite there being only a very few amphibian species resident in Israel, there is still no consensus among zoologists regarding the exact number of species inhabiting this small country, or their geographical distribution. Until recently, seven amphibian species were considered monotypic^7^, i.e. they belong to only distantly related genera. One of these species is the Middle East tree frog (*Hyla savignyi* Audouin, 1827). In 2007, Grach et al.^8^ described a new endemic tree frog species (*H. heinzsteinitzi* Grach, Plessed & Werner, 2007) from Jerusalem and the adjacent Judean Hills on the basis of morphological and bioacoustics data. However, as their work was not corroborated by molecular analyses, their results were largely criticized. Stöck et al.^9^, who investigated two mitochondrial DNA fragments (cytochrome oxidase subunit I gene and cytochrome* b*) of a single sample from the type locality in Jerusalem and compared the provided morphological and acoustic data of *H. heinzsteinitzi* with those of other *Hyla* species, suggested that the localized population of *H. heinzsteinitzi* might be the result of a human introduction of another species [*H. japonica* (Günther, 1859)] into Israel. However, their conclusion was based solely on the sequences of a single individual found in GenBank, which was again heavily criticized by Werner^10^. In their study, which sought to resolve the phylogeny of circum-Mediterranean tree frogs, Stöck et al.^9^ nonetheless also revealed that the then single nominal and widespread species *H. savignyi* (distribution range: Cyprus, Egypt, Georgia, Iran, Iraq, Israel, Jordan, Lebanon, Syria, and Turkey) in fact comprises two evolutionarily distinct lineages that deserve the status of separate species^9^*.* This finding was supported by another study that finally divided the nominal species into a northern clade, for which the name *H. savignyi* was kept; and a southern clade, now known as *H. felixarabica* Gvoždík, Kotlík & Moravec, 2010^11^. This latter study included 15 samples from Israel and suggested that both species are present in the country. This finding, however, was not accepted by Degani et al.^12^, who did not differentiate between *H. savignyi* and *H. felixarabica* in their study, in which they investigated the variation in *Hyla* populations in northern Israel. In two studies from 2019 and 2020, Dufresnes et al.^13,14^ confirmed the hypothesis of Gvoždík et al.^11^. However, Dufresnes et al.’s studies were largely based on the same samples as those of Gvoždík et al., adding 12 more individuals from two additional pools in Israel. Therefore, the exact distribution of each of these two species in Israel, as well as the taxonomic validity of *H. heinzsteinitzi* have remained unclear.

Here, we sought to resolve the taxonomic status of frogs belonging to the genus *Hyla* in Israel. Based on an extensive sampling of freshwater habitats throughout the country, we attempted to elucidate the geographic distribution for each of the known species, and to investigate the presence of potential additional species or of significant evolutionary units in this region. We also provide information on the divergence between the different species and identify barriers to mitochondrial gene flow.

## Results

**Figure 1:**
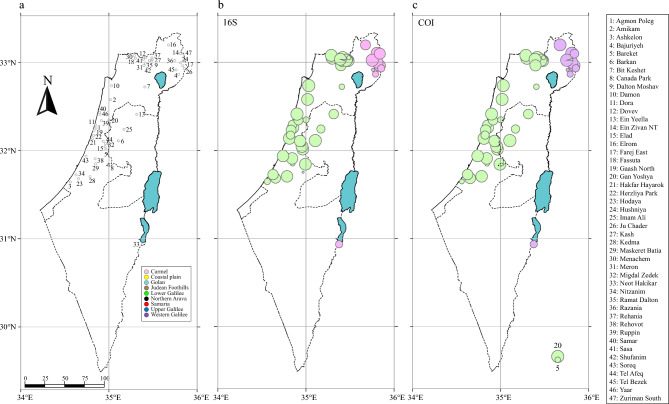
(**a**) Map showing the locations of the 47 sampled sites across Israel. Number of individuals of *Hyla savignyi* (green circles) and *H. felixarabica* (pink circles) sampled at each locality based on mtDNA 16S (**b**) and COI (**c**). Circle sizes represent sample sizes.

**Figure 2:**
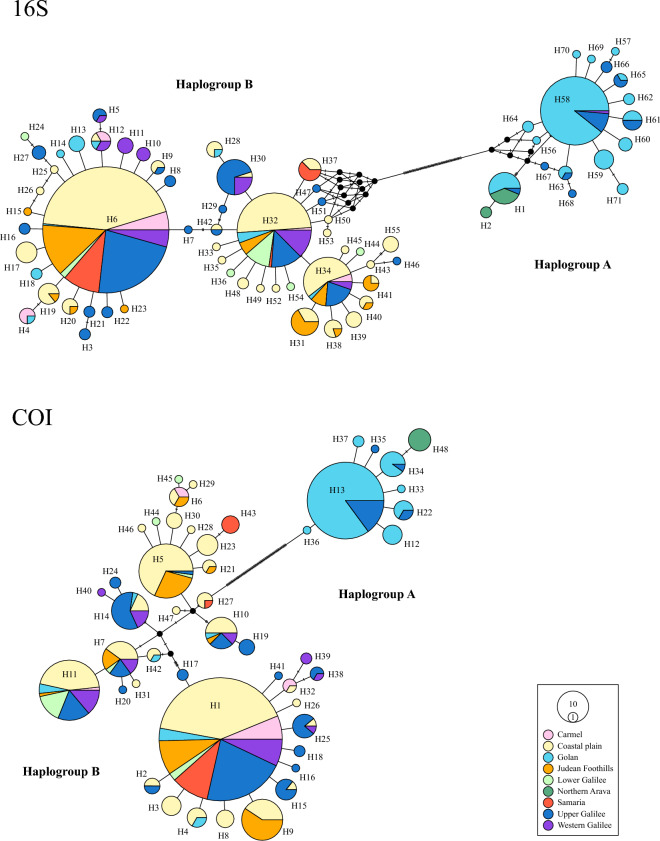
Median-joining haplotype networks of 16S and COI haplotypes of *Hyla* individuals collected from nine regions across Israel. Circle sizes represent haplotype frequencies, cross bars between haplotypes denote one mutation each, and colors correspond to the different regions. Branches are not to scale.

In this study, we analyzed 16S rRNA and COI gene fragments of 658 *Hyla* individuals sampled at 47 rain pools in nine regions across Israel and the West Bank (hereafter combined as ‘Israel’; Fig. 1a). Our genetic diversity analyses revealed 109 polymorphic sites resulting in 71 haplotypes for 16S; and 102 polymorphic sites resulting in 48 haplotypes for COI. The overall haplotype diversity was relatively high for both gene fragments, being slightly higher for COI (16S: *Hd* = 0.815 ± 0.013, *π* = 0.021; COI: *Hd* = 0.828 ± 0.012, *π* = 0.044). The high diversity was also reflected in our median-joining haplotype networks, which revealed two main distinct haplogroups (A and B) for each gene fragment that were separated from one another by at least 63 (16S) and 64 (COI) mutational steps, respectively (Fig. 2). For both gene fragments, Haplogroup A was smaller (16S: 18 haplotypes; COI: 9 haplotypes) and was only found in individuals from three out of the nine regions (Golan, northern Arava, and Upper Galilee). In this haplogroup, individuals from one location (northern Arava) not only stood out in possessing a unique haplotype for each gene fragment, but also in the overall low number of haplotypes (16S: 2 haplotypes; COI: 1 haplotype; Fig. 2). By contrast, Haplogroup B was larger (16S: 53 haplotypes; COI: 39 haplotypes) and far more diverse. For each gene fragment, three haplotypes were shared by numerous individuals from several regions (16S: H6 = 252 individuals from 8 regions, H32 = 87 individuals from 8 regions, H34 = 38 individuals from 5 regions; COI: H1 = 241 individuals from 8 regions, H5 = 58 individuals from 4 regions, H11 = 94 individuals from 7 regions; Fig. 2). However, there were also several haplotypes for each gene fragment that were represented by only one or two of the examined individuals (Fig. 2).

**Figure 3:**
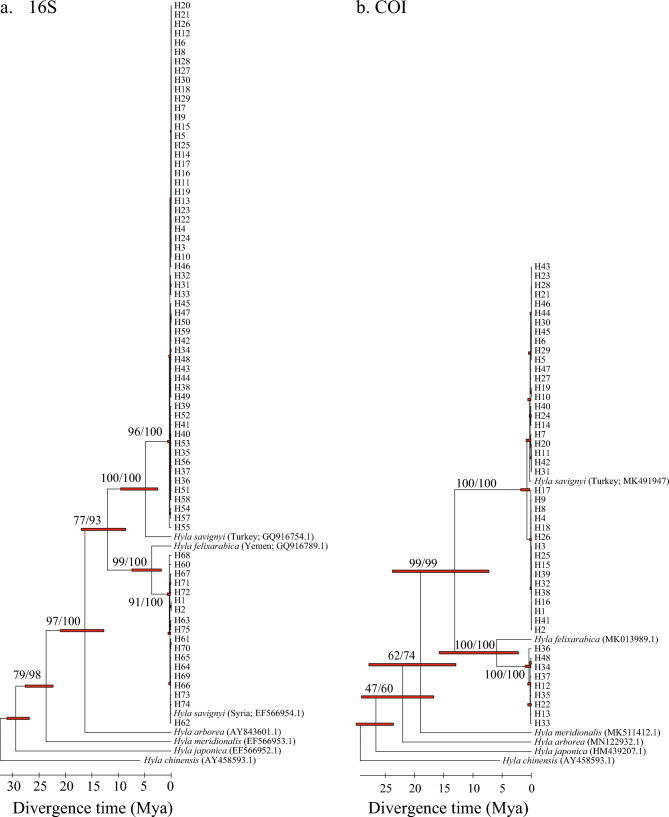
Time-trees for the haplotypes of 658 *Hyla* individuals from Israel as well as outgroups based on the ML phylogenetic tree output for (**a**) a 16S rRNA gene fragment and (**b**) a COI fragment. Estimated upper and lower ranges for each node are represented as red bars. Timescale in million years. Note that for 16S the *H. savignyi* sequences obtained from GenBank are of different origins. While one sequence was obtained from an individual sampled in Turkey, another sequence was obtained from an individual sampled in Syria prior to the study, classifying individuals from southern Syria as *H. felixarabica*^10^.

Our time-tree analyses based on the obtained haplotypes for each gene fragment yielded very similar topologies as recovered from the Bayesian and ML analyses, which revealed two clades (A and B) that probably diverged between the early Pliocene and late Miocene. Overall, the calculated times of divergence were similar for both gene fragments. For the 16S rRNA gene fragment, the time of divergence between the two clades was dated to 12.1 Mya (95% CI: 8.9 and 17.1 Mya; Fig. 3a), while for the COI gene fragment the divergence was dated to 13.1 Mya (95% CI: 7.1 and 23.6 Mya; Fig. 3b).

### Population structure and demographic analyses

**Figure 4:**
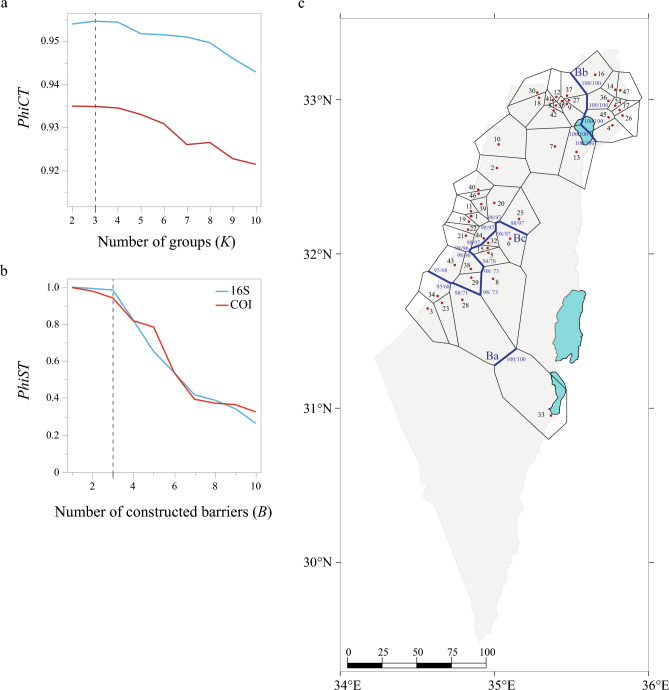
Structure clustering results of 47 *Hyla* populations throughout Israel based on two gene fragments. (**a**) *PhiCT* values for groups (*K*) 2 to 10 as obtained by SAMOVA analyses, and (**b**) *PhiST* values for constructed barriers (*B*) 1 to 10 as obtained by the Monmonier algorithm. Broken vertical line indicates the most likely number of *K* and *B*. (**c**) Geographical barrier test (Monmonier’s algorithm) for 47 *Hyla* populations throughout Israel based on two gene fragments (16S rRNA gene and COI). Populations are represented by a red dot and a corresponding black ID number (For details see Table S2); black thin lines depict the Voronoï tessellation of the populations according to their geographic locations; and blue lines constitute the three main genetic barriers (*Ba*, *Bb* and *Bc*) detected through a bootstrap analysis based on 100 permutations. Blue numbers indicate the bootstrap support for 16S/COI.

For each gene fragment, we discovered significant global genetic differentiation using the genetic distance *G*_*ST*_ (16S: 0.323, *P* < 0.0001; COI: 0.323, *P* < 0.0001), *N*_*ST*_ (16S: 0.910, *P* < 0.0001; COI: 0.874, *P* < 0.0001) and *PhiST* (16S: 0.897, *P* < 0.0001; COI: 0.859, *P* < 0.0001). We found significantly larger *N*_*ST*_ than* G*_*ST*_ values (16S: 0.587, *P* < 0.0001; COI: 0.551, *P* < 0.0001), which suggests a strong phylogeographic structuring. For the 16S fragment, the SAMOVA analysis partitioned the 47 sampled populations into three groups (*K* = 3) while revealing almost identical *PhiCT* values for *K* = 2 and *K* = 3 for the COI fragment (Table 1, Fig. 4a). We found the population of the northern Arava (Neot Hakikar) to be the cause of this ambiguous result, as this population formed a clearly separate ‘group’ for 16S, but not for COI. We therefore adopted the three-group solution (for a list of populations in these groups see Table S1). Post-hoc AMOVA statistics on *K* = *3* as partitioned by SAMOVA were all found to be significant (*P* < 0.0001) for each locus. Values were high for both *PhiCT* values (genetic variance among groups: 16S = 0.92; COI = 0.90) and *PhiST* values (genetic variance within populations: 16S = 0.96; COI = 0.95), with the latter indicating a high number of private haplotypes, as corroborated by the haplotype network (Fig. 2). By contrast, the *PhiSC* values (genetic variance among populations within groups) were comparatively low (16S: *PhiSC* = 0.52; COI: *PhiSC* = 0.46). Pairwise AMOVA *PhiST* confirmed a clear differentiation among the three groups. All pairwise *PhiST* values were significant (*P* < 0.0001), ranging from 0.586 to 0.925, suggesting no or extremely limited mitochondrial gene flow between the different population clusters. Interestingly, the population of the northern Arava (Group 1) showed more similarity to that of the Golan region (Group 2) than to the geographically closer populations of Group 3 (AMOVA *PhiST* values for the 16S and the COI fragment: Group 1 – Group 2 = 0.586 and 0.598, respectively; Group 1 – Group 3 = 0.923 and 0.898, respectively; Group 2 – Group 3 = 0.925 and 0.906, respectively; all values were significant *P* < 0.0001). Negative values of neutrality tests (Tajima’s *D* and Fu’s *F*_*S*_) performed on each of the groups indicated either selection removing variation or a recent population expansion for at least two of the groups (Table 2).

**Table 1:** Results of the SAMOVA analyses. The highest *PhiCT* value (in bold) indicates the optimal cluster solution (optimal number of *K*).

No. of *K*	*PhiCT* value	
	16S	COI
1	0.8969	0.8594
2	0.9540	**0.9349**
3	**0.9546**	0.9348
4	0.9544	0.9345
5	0.9517	0.9330
6	0.9515	0.9308
7	0.9509	0.9260
8	0.9496	0.9265
9	0.9460	0.9227
10	0.9428	0.9214

**Table 2:** Summary statistics and results of the neutrality tests (Tajima's *D* and Fu's *FS* tests) for the three groups identified by SAMOVA.

		Group 1	Group 2	Group 3
16S	Nh:	2	17	57
	Xh:	0.028	0.239	0.803
	Pi:	0.000	0.002	0.005
	Pir:	0.021	0.356	1.882
	Ar:	2	3.747	4.490
	Tajima's *D*	0.334	− **2.672**	− 1.590
	(*P*-value)	(> 0.10)	(< 0.01)	(0.10)
	Fu's *F*_*S*_	0.536	− 6.009	− 32.259
COI	Nh:	1	9	41
	Xh:	0.021	0.188	0.854
	Pi:	0	0.003	0.013
	Pir:	0	0.196	3.689
	Ar:	1	2.541	4.778
	Tajima's *D*	n.a	− **2.792**	− 0.548
	(*P*-value)	n.a	(< 0.001)	(> 0.10)
	Fu's *F*_*S*_	n.a	− 0.507	− 6.772

Significant values are in bold. Nh: total number of haplotypes; Xh: ratio between the number of haplotypes in the defined group of populations and the total number of haplotypes; Pi: nucleotide diversity; Pir: ratio between the nucleotide diversity within the defined group of populations and the nucleotide diversity of all other populations; Ar: allelic richness. For details on groups see Table S1.

A Monmonier algorithm identified three potential barriers (*Ba*, *Bb,* and *Bc*) between four different population clusters, suggesting limited mtDNA gene flow or even genetic isolation between them (Fig. 4b,c). The locations of the computed barriers were identical between the two gene fragments, but their robustness was only congruent for two out of the three barriers. Barrier *Ba*, which separates the single population of the northern Arava (Neot Hakikar) from the closest bordering population in the Coastal Plain (Kedma), received 100% bootstrap support for both gene fragments. Barrier *Bb*, separating the nine populations of the Golan region (Bajuriyeh, Ein Zivan NT, Elrom, Farej East, Hushniya, Juchader, Razania, Tel Bezek, Zuriman South) as well as a single population of the Lower Galilee (Ein Yeella) from the bordering populations, likewise received 100% bootstrap support for both gene fragments. Barrier *Bc*, running between the populations of Samaria (Barkan and Imam Ali) and separating the populations of the Judean foothills (Bareket and Canada Park) as well as the southern populations of the Coastal Plain (Ashkelon, Elad, Hodaya, Kedma, Migdal Zedek and Nitzanim) from the other populations of the Coastal Plain, was weaker and only received a bootstrap support of 54–98% (16S) and 68–97% (COI) (Fig. 4c).

## Discussion

Our collective results based on DNA sequence data corroborate previous reports on two different cryptic *Hyla* species in Israel^9,11,13^. Our generated trees display a clear division into two main clades (A and B), which strictly conform to haplogroups A and B in the haplotype network (Fig. 2). We identified the two clades as corresponding to the species *H. felixarabica* (clade A) and *H. savignyi* (clade B), thus ruling out their identification as a single species, as suggested by Degani et al.^12^. However, it should be noted that while Degani et al. used the two species’ names interchangeably, their study was restricted to the Upper Galilee, and thus the individuals they investigated must have exclusively belonged to *H. savignyi.*

We found the *Hyla savignyi* sequences obtained from GenBank clustering within both clades for the 16S rRNA gene fragment, and within clade A for the COI gene fragment. While these results initially seemed contradictory, one 16S sequence was in fact obtained from an individual that had been captured in Syria prior to the study by Gvoždík et al.^11^, in which individuals from southern Syria were classified as belonging to *H. felixarabica*. Even though we do not know where in Syria this individual was sampled, our combined data suggest that the 16S sequence does indeed correspond to *H. felixarabica.* We did not find any genetic support for the existence of *H. heinzsteinitzi* previously described by Grach et al.^8^, although it should be noted that the original location of the putative species has been functionally destroyed. For roughly the past decade, this water body has not carried enough water to support amphibian breeding and has also been heavily sprayed with pesticides (S.G., pers. obs.). Despite multiple visits during our study period, the site was mostly completely dry and we were unable to detect adults or tadpoles of any frog species at this location or in its vicinity.

In accordance with previous studies, we found the *Hyla felixarabica* clade to be distributed east and south of the Dead Sea Rift (Golan and northern Arava), while the *H. savignyi* clade is only distributed west of the Dead Sea Rift (all other regions)^11,13,14^. Consistent with the calculations by Gvoždík et al.^11^ as well as Li et al.^15^, who presented a phylogeny including almost all described *Hyla* species, our time-tree results estimate the division between *H. felixarabica* and *H. savignyi* to have occurred between the Early Pliocene and Late Miocene, when the local plate kinematics still formed the Dead Sea Rift^16,17^. Only at one location from the Golan region (Razania), did we identify one individual as belonging to *H. savignyi*. The other 19 individuals captured at this pool clustered as *H. felixarabica*. According to Dufresnes et al.^13^, *H. felixarabica* and *H. savignyi* are parapatric along their contact zone of hundreds of kilometers. While we acknowledge that we only sampled a maximum of 20 individuals per site, our observations revealed an almost exclusively allopatric distribution of these two species. Even though we captured a single *H. savignyi* individual at the Razania pool, we cannot rule out the possibility that this individual had been accidentally translocated to Razania from a location west of the Dead Sea Rift (e.g. via a delivery of plants, fruits, or vegetables; Fig. 1). It is not unlikely that *H. felixarabica* and *H. savignyi* have evolved different habitat requirements over the past million years, which could explain why these two species are not commonly found at the same locality. Hosseinian-Yousefkhani et al.^18^ modeled the potential distribution areas based on capture data and bioclimatic variables for three hylid species occurring in the Middle East and revealed that *H. felixarabica* is restricted to only a few arid regions along the eastern side of the Rift Valley, while *H. savignyi* is widely distributed in semi-arid habitats^18^. By contrast, Dufresnes et al.^13^ conducted a similar analysis, limited to *H. felixarabica* and *H. savignyi*, and found no clear indications of differential adaptations to climatic conditions between the two species. Rather, they hypothesized that the species’ dispersal is more impeded by the barren landscape in this region than by the differences in climatic conditions. We would like to present an alternative hypothesis, which is based on the fact that the divergence time between *H. felixarabica* and *H. savignyi* is very long (5.2 mya ^14^, 12.5 mya this study). The dispersal of populations north of the Dead Sea on both side of the Rift Valley is not restricted, because individuals can move freely through the major tributaries of the Jordan River, which are wet year round. By contrast, populations of *H. felixarabica* south of the Dead Sea and along the western coast of South Arabia, including Yemen, are isolated to oases or wadis with permanent water flow. These populations are indeed isolated by barren and extreme arid desert. However, South Arabia has not always been an arid desert. This region is influenced by the Saharan hydroclimate, which is characterized by an alternation of prolonged dry and wet periods at intervals of ~ 10,000 years. These African humid periods (AHPs) extended back to at least 10 million years BP, and were identified to occur ~ 230 times in the past 8 million years^19^. During peaks of AHPs, climatic conditions and vegetation patterns were probably more favorable for the dispersal of amphibians in South Arabia, and the native *H. felixarabica* could have migrated from Yemen northwards to Jordan and Syria. Such a scenario could explain the deep divergence time between *H. felixarabica* and *H. savignyi*. We suggest that a genetic study on a more extensive sampling of *H. felixarabica* from South Arabia is required for testing the above hypothesis.

Even though our combined results suggest that there are only two *Hyla* species in Israel, limits to gene flow were also detected between populations of each species, as reflected in the high number of unique haplotypes distributed across the different regions. This was also supported by the identified barrier running across the center of Israel and separating the northern from the southern *H. savignyi* populations (*Bc*)*.* In addition, our analyses partitioned the 47 populations into three groups, with the single population in the northern Arava (Neot Hakikar), which phylogenetically belongs to *H. felixarabica*, forming its own group*.* The sampled individuals of this population were captured in wetlands bordering parts of the southern remnant of the Dead Sea (salt evaporation ponds) in the surroundings of Neot Hakikar, an agricultural community on its southern tip. This community is located at the border with Jordan within the outskirts of the nearest Jordanian village, Fifa, only a few hundred meters away. Even though there appear to be no current admixture events between the population at Neot Hakikar and any other *Hyla* population in Israel, it is very likely that this population extends further into the wetlands around Fifa, from where it probably originates. Unfortunately, formerly extensive wetlands in both Israel and Jordan^20^ have been largely reduced due to agricultural and industrial developments^21–24^. Following the construction and growth of settlements such as Neot Hakikar, water has become more extensively used for industry and agriculture^25–27^. Over the past decade, the Tamar Regional Council (the official municipality of this region on the Israeli side) has been converting several locations into unprotected eco-parks simulating the former moist habitat in the area. On the Jordanian side, efforts to protect the former wetlands have proven more fruitful. In 2011, the Fifa Nature Reserve was established by the Jordanian Royal Society for Conservation of Nature (RSCN)^28^. This reserve was designated the world’s lowest elevation Ramsar Site in 2017 (426 m below sea level)^29^. However, both the regional parks around Tamar, as well as the Fifa Reserve, are very small areas compared to the original wetlands.

Against the backdrop of global climate change and concomitantly changing weather regimes, the limited gene flow between the different *Hyla* populations of the same species in Israel is disquieting. We strongly recommend maintaining and improving the connectivity between these different localities where possible in order to maintain healthy and adaptable species. We further urge Israeli decision-makers, particularly in the northern Arava region, to improve and advance protection of the wetland habitat around Neot Hakikar, and to extend it as far as possible to the border with Jordan. We found that the *Hyla* population in this area formed its own unique cluster and, even though we only managed to capture eight *Hyla* individuals at this pool, our results suggest that this population may qualify as a separate evolutionarily significant unit. Finally, we also encourage Jordan to further extend their wetland habitat to the border with Israel and conduct further population genetic studies on *Hyla* on the Jordanian side in order to confirm this population’s unique status and promote its protection.

## Methods

### Sample collection and processing

We obtained DNA from (a) tissue samples of ethanol-preserved specimens previously collected by Yishai Weisman between March and June 2012; and (b) toe clips or buccal swabs taken from adults, and tail clips taken from tadpoles collected in the field during multiple sampling trips conducted between March 2019 and June 2020 and stored in ethanol until further processing. Live individuals were immediately released to the wild after tissue sampling. Altogether, we extracted the total genomic DNA of 658 individuals from 47 locations within nine geographic regions of Israel and the West Bank (Fig. 1a, Table S2) using the AccuPrep Genomic DNA Extraction Kit (BioNeer, Korea) for tissue samples and the DNeasy Blood & Tissue Kit (Qiagen, Germany) for swab samples, in accordance with the manufacturers’ instructions.

We PCR amplified a 1,003 base pairs (bp) fragment of the 16S rRNA gene (16S) using the primer pair 16SIschF1 and 16SIschR1 (designed by M. Gehara), and a 577 bp fragment of the cytochrome oxidase subunit I gene (COI) using the primer pair COIVertF1 and COIVertR1^30^. The thermocycling profile for 16S comprised an initial denaturation step at 94 °C for 90 s, followed by 35 cycles of denaturation (94 °C for 45 s), annealing (55 °C for 45 s) and elongation (72 °C for 90 s), and a final elongation step at 72 °C for 10 min. For COI, the thermocycling profile comprised an initial denaturation step at 94 °C for 2:20 min, followed by 35 cycles of denaturation (94 °C for 30 s), annealing (48 °C for 45 s) and elongation (72 °C for 90 s), and a final elongation step at 72 °C for 10 min. We visualized the PCR products on 2% agarose gels and determined the DNA concentration using a NanoDrop One C spectrophotometer (Thermo Fisher Scientific, USA). Subsequently, the products were sent to a commercial laboratory (MCLAB, San Francisco, USA; https://www.mclab.com) for purification and Sanger sequencing on ABI 3730XL sequencers (Applied Biosystems, USA).

### Sequence alignment and basic statistical analyses

We checked and trimmed the sequences using CodonCode Aligner version 9.0.2 (CodonCode Corporation, Dedham, MA, USA) and verified sequence identity using the BLAST tool of NCBI (https://blast.ncbi.nlm.nih.gov/Blast.cgi). We then constructed a map for each gene fragment displaying the number of individuals per species for each site (Fig. 1b,c) using ArcGIS version 10 (ESRI Inc.). Next, we aligned the sequences in MEGA X 10.2.6^31^ using the implemented ClustalW algorithm.

We collapsed identical haplotypes for each mitochondrial fragment, and calculated haplotype (*Hd*) and nucleotide (*π)* diversity as well as the number of polymorphic sites for both markers using DnaSP 6.12^32^. To visualize the evolutionary relationships between populations, we constructed an unrooted median-joining haplotype network^33^ for each gene fragment in PopART 1.7^34^.

### Phylogenetic analyses and estimates of divergence time

For the phylogenetic analyses, we added published sequences of *H. savignyi* and *H. felixarabica* as well as additional outgroup species (*H. arborea*; *H. meridionalis*; *H. japonica* and *H. chinensis*) to the alignments to reduce uncertainty on node ages (Table S3). Based on the obtained haplotype sequences, we generated separate phylogenetic trees for each gene fragment under both a Bayesian framework in MrBayes 3.2.7^35,36^, and a Maximum Likelihood (ML) framework in MEGA X^37^. The ‘best model test’, as implemented in MEGA X, identified the Tamura Nei (1993; TN + G) gamma and the Hasegawa-Kishino-Yano (1985; HKY +) invariant sites models of nucleotide substitution^38,39^ as the best fit for the 16S and COI data sets, respectively. For the Bayesian analyses, we ran two independent *Markov chain Monte Carlo* (MCMC) runs starting from random trees with three hot chains and one cold chain each. Trees were sampled every 1,000 generations for a total of 10,000,000 generations, and 25% of generations were discarded as burn-in. Subsequently, we used Tracer v. 1.7.2^40^ to check the chains for convergence. For the ML analyses, we used a BioNJ starting tree with a Nearest-Neighbor-Interchange (NNI) tree topology search^41^. Robustness of ML tree topologies were tested by bootstrap analyses^42^ with 1,000 replicates each. Lastly, to provide an historical context of sequence variation, we constructed time-calibrated trees (time-trees) based on the ML phylogenetic tree output using the robust RelTime-ML method^43^, which computes estimates based on branch lengths optimized by ML, and divergence time calibration as implemented in MEGA X^44^. We used the divergence time confidence intervals between *H. meridionalis* and *H. japonica* (22.07–31.17 Mya; Time-tree database incorporated in MEGA X [add constraints/uniform distribution]) to calibrate divergence time of all nodes on both gene trees.

### Population structure and demographic analyses

For each gene fragment, we investigated the global genetic differentiation by determining *N*_*ST*_, *G*_*ST*_^45,46^ and *PhiST*^47^ values based on 10,000 random permutations using SPADS 1.0^48^. We further calculated the difference between *N*_*ST*_ and *G*_*ST*_ values to test the strength of the phylogeographic signal. In SPADS, we also applied two clustering algorithms to study the genetic structure among populations: (i) a spatial analysis of molecular variance (SAMOVA) aimed at identifying geographically homogeneous and maximally differentiated population groups^49^; and (ii) Monmonier’s maximum-difference algorithm that identifies zones of distinct genetic boundaries (genetic barriers) between sampled locations^49,50^. SAMOVA analyses were performed with putative numbers of populations (*K*) ranging from 2 to 10 with 10,000 iterations applied in 10 replicate runs, and we chose the optimum value for *K* based on the highest *PhiCT* value. We then ran analyses of molecular variance (AMOVAs) on the groups (*K*) as delineated by SAMOVA, and determined AMOVA *PhiST* values based on 10,000 random permutations. Subsequently, we used DnaSP to investigate past changes of effective population size between the groups as identified by SAMOVA by calculating neutrality statistics with Tajima's *D*^51^ and Fu's *F*_*S*_^52^. We further performed the Monmonier algorithm with 100 bootstrapped matrices to assess robustness and putative numbers of barriers (*B*) ranging from 1 to 10. Lastly, we visualized the polygonal neighborhood for each population (Voronoï tessellation) and computed barriers among populations using BARRIER 2.2^53^.

### Supplementary Information

Supplementary Information.

## Data Availability

The datasets generated and analyzed in this study are available in the GenBank repository with accession numbers OR016783–OR017108, OR017110–OR017418, OR017420–OR017231, OR017233–OR017443 [16S]; OR017449–OR017523, OR017525–OR017530, OR017532–OR017562, OR017564–OR018109 [COI].
