# Supplementary material for: The phylogeography of Middle Eastern tree frogs in Israel
Source: Sci Rep. 2024 Feb 2;14:2788. doi: 10.1038/s41598-024-52700-5 (PMC10837426; doi:10.1038/s41598-024-52700-5)
Supplement: Supplementary file 1 — Supplementary Information. [file 41598_2024_52700_MOESM1_ESM.pdf]

Supplementary Information

The phylogeography of Middle Eastern tree frogs in Israel

Gal Mesika Surizon, Eli Geffen, Uri Roll, Sarig Gafny, R. G. Bina Perl

Supplementary Table S1. List of populations included in each of the three groups as partitioned by SAMOVA.

	# of Pop.	Populations
Group 1	1	Neot Hakikar
Group 2	9	Bajuriyeh, Ein Zivan NT, Elrom, Farej East, Hushniya, Juchader, Razania, Tel Bezek, Zuriman South
Group 3	37	Agmon Poleg, Amikam, Ashkelon, Bareket, Barkan, Bit Keshet, Canada Park, Dalton Moshav, Damon, Dora, Dovev, Ein Yeella, Elad, Fassuta, Gaash North, Gan Yoshya, Hakfar Hayarok, Herzliya Park, Hodaya, Imam Ali, Kash, Kedma, Maskeret Batia, Menachem, Meron, Migdal Zedek, Nitzanim, Ramat Dalton, Rehania, Rehovot, Ruppin, Samar, Sasa, Shufanim, Soreq, Tel Afeq, Yaar

Supplementary Table S2. List of individuals sampled in this study.

Date	Sample ID	Individual ID	Life Stage	Type of Sample	Population No.	Pool	Coordinates	GenBank accession number (16S)	GenBank accession number (COI)
16.02.2020	GMS2019-024	#024	Tadpole	Tail clip	34	Nitzanim	31.72422, 34.62641	OR016996	OR017449
16.02.2020	GMS2019-026	#026	Tadpole	Tail clip	34	Nitzanim	31.72422, 34.62641	OR017050	OR017450
16.02.2020	GMS2019-028	#028	Tadpole	Tail clip	34	Nitzanim	31.72422, 34.62641	OR016995	OR017451
16.02.2020	GMS2019-029	#029	Tadpole	Tail clip	34	Nitzanim	31.72422, 34.62641	OR017049	OR017452
16.02.2020	GMS2019-030	#030	Tadpole	Tail clip	34	Nitzanim	31.72422, 34.62641	OR017048	OR017453
16.02.2020	GMS2019-031	#031	Tadpole	Tail clip	34	Nitzanim	31.72422, 34.62641	OR017047	OR017454
16.02.2020	GMS2019-032	#032	Tadpole	Tail clip	34	Nitzanim	31.72422, 34.62641	OR016994	OR017455
16.02.2020	GMS2019-034	#034	Tadpole	Tail clip	34	Nitzanim	31.72422, 34.62641	OR017300	OR017456
16.02.2020	GMS2019-035	#035	Tadpole	Tail clip	34	Nitzanim	31.72422, 34.62641	OR016993	OR017457
16.02.2020	GMS2019-036	#036	Tadpole	Tail clip	34	Nitzanim	31.72422, 34.62641	OR017274	OR017458
16.02.2020	GMS2019-038	#038	Tadpole	Tail clip	34	Nitzanim	31.72422, 34.62641	OR017046	OR017459
16.02.2020	GMS2019-039	#039	Tadpole	Tail clip	34	Nitzanim	31.72422, 34.62641	OR017045	OR017460
16.02.2020	GMS2019-042	#042	Tadpole	Tail clip	34	Nitzanim	31.72422, 34.62641	OR017313	OR017461
16.02.2020	GMS2019-044	#044	Tadpole	Tail clip	34	Nitzanim	31.72422, 34.62641	OR016992	OR017462
16.02.2020	GMS2019-047	#047	Tadpole	Tail clip	34	Nitzanim	31.72422, 34.62641	OR017044	OR017463
16.02.2020	GMS2019-049	#049	Tadpole	Tail clip	3	Ashkelon	31.6451938, 34.5614702	OR017084	OR017464
16.02.2020	GMS2019-050	#050	Tadpole	Tail clip	23	Hodaya	31.68215, 34.65542	OR017043	OR017465
16.02.2020	GMS2019-051	#051	Tadpole	Tail clip	23	Hodaya	31.68215, 34.65542	OR017042	OR017466
16.02.2020	GMS2019-054	#054	Tadpole	Tail clip	23	Hodaya	31.68215, 34.65542	OR017041	OR017467
16.02.2020	GMS2019-055	#055	Tadpole	Tail clip	23	Hodaya	31.68215, 34.65542	OR017040	OR017468
16.02.2020	GMS2019-056	#056	Tadpole	Tail clip	23	Hodaya	31.68215, 34.65542	OR016991	OR017469
16.02.2020	GMS2019-057	#057	Tadpole	Tail clip	23	Hodaya	31.68215, 34.65542	OR017235	OR017470
16.02.2020	GMS2019-058	#058	Tadpole	Tail clip	23	Hodaya	31.68215, 34.65542	OR017039	OR017471
16.02.2020	GMS2019-061	#061	Tadpole	Tail clip	23	Hodaya	31.68215, 34.65542	OR017038	OR017472
16.02.2020	GMS2019-062	#062	Tadpole	Tail clip	23	Hodaya	31.68215, 34.65542	OR017093	OR017473

Date	Sample ID	Individual ID	Life Stage	Type of Sample	Population No.	Pool	Coordinates	GenBank accession number (16S)	GenBank accession number (COI)
16.02.2020	GMS2019-063	#063	Tadpole	Tail clip	23	Hodaya	31.68215, 34.65542	OR017037	OR017474
16.02.2020	GMS2019-065	#065	Tadpole	Tail clip	23	Hodaya	31.68215, 34.65542	OR017036	OR017475
16.02.2020	GMS2019-070	#070	Tadpole	Tail clip	23	Hodaya	31.68215, 34.65542	OR017035	OR017476
16.02.2020	GMS2019-071	#071	Tadpole	Tail clip	23	Hodaya	31.68215, 34.65542	OR017034	OR017477
16.02.2020	GMS2019-072	#072	Tadpole	Tail clip	23	Hodaya	31.68215, 34.65542	OR017033	OR017478
27.02.2020	GMS2019-073	#073	Adult	Toe clip	3	Ashkelon	31.6451938, 34.5614702	OR017083	OR017479
27.02.2020	GMS2019-074	#074	Adult	Toe clip	3	Ashkelon	31.6451938, 34.5614702	OR017082	OR017480
27.02.2020	GMS2019-075	#075	Adult	Toe clip	3	Ashkelon	31.6451938, 34.5614702	OR017081	OR017481
27.02.2020	GMS2019-076	#076	Adult	Toe clip	3	Ashkelon	31.6451938, 34.5614702	OR017080	OR017482
27.02.2020	GMS2019-077	#077	Adult	Toe clip	3	Ashkelon	31.6451938, 34.5614702	OR017079	OR017483
05.03.2020	GMS2019-079	#079	Tadpole	Tail clip	28	Kedma	31.7039114, 34.7852847	OR017078	OR017484
05.03.2020	GMS2019-080	#080	Tadpole	Tail clip	28	Kedma	31.7039114, 34.7852847	OR017273	OR017485
05.03.2020	GMS2019-081	#081	Tadpole	Tail clip	28	Kedma	31.7039114, 34.7852847	OR017234	OR017486
05.03.2020	GMS2019-083	#083	Tadpole	Tail clip	28	Kedma	31.7039114, 34.7852847	OR017032	OR017487
05.03.2020	GMS2019-084	#084	Tadpole	Tail clip	28	Kedma	31.7039114, 34.7852847	OR017233	OR017488
05.03.2020	GMS2019-085	#085	Tadpole	Tail clip	28	Kedma	31.7039114, 34.7852847	OR017031	OR017489
05.03.2020	GMS2019-086	#086	Tadpole	Tail clip	28	Kedma	31.7039114, 34.7852847	OR017030	OR017490
05.03.2020	GMS2019-087	#087	Tadpole	Tail clip	28	Kedma	31.7039114, 34.7852847	OR017029	OR017491
05.03.2020	GMS2019-088	#088	Tadpole	Tail clip	28	Kedma	31.7039114, 34.7852847	OR017028	OR017492
05.03.2020	GMS2019-089	#089	Tadpole	Tail clip	28	Kedma	31.7039114, 34.7852847	OR017027	OR017493
05.03.2020	GMS2019-090	#090	Tadpole	Tail clip	28	Kedma	31.7039114, 34.7852847	OR017026	OR017494
05.03.2020	GMS2019-091	#091	Tadpole	Tail clip	28	Kedma	31.7039114, 34.7852847	OR017025	OR017495
05.03.2020	GMS2019-092	#092	Tadpole	Tail clip	28	Kedma	31.7039114, 34.7852847	OR017024	OR017496
05.03.2020	GMS2019-093	#093	Tadpole	Tail clip	28	Kedma	31.7039114, 34.7852847	OR017023	OR017497
05.03.2020	GMS2019-095	#095	Tadpole	Tail clip	28	Kedma	31.7039114, 34.7852847	OR017022	OR017498
05.03.2020	GMS2019-096	#096	Tadpole	Tail clip	28	Kedma	31.7039114, 34.7852847	OR017021	OR017499
05.03.2020	GMS2019-099	#099	Tadpole	Tail clip	28	Kedma	31.7039114, 34.7852847	OR017020	OR017500

Date	Sample ID	Individual ID	Life Stage	Type of Sample	Population No.	Pool	Coordinates	GenBank accession number (16S)	GenBank accession number (COI)
05.03.2020	GMS2019-100	#100	Tadpole	Tail clip	28	Kedma	31.7039114, 34.7852847	OR017019	OR017501
04.07.2019	GMS2019-101	#101	Tadpole	Tail clip	47	Zuriman South	33.09659, 35.83913	OR017409	OR017502
04.07.2019	GMS2019-103	#103	Tadpole	Tail clip	47	Zuriman South	33.09659, 35.83913	OR017432	OR017503
04.07.2019	GMS2019-104	#104	Tadpole	Tail clip	47	Zuriman South	33.09659, 35.83913	OR017408	OR017504
24.04.2019	GMS2019-117	#117	Tadpole	Tail clip	40	Samar	32.43455, 34.89416	OR016990	OR017505
24.04.2019	GMS2019-120	#120	Tadpole	Tail clip	40	Samar	32.43455, 34.89416	OR017018	OR017506
24.04.2019	GMS2019-121	#121	Tadpole	Tail clip	40	Samar	32.43455, 34.89416	OR017056	OR017507
24.04.2019	GMS2019-122	#122	Tadpole	Tail clip	40	Samar	32.43455, 34.89416	OR017113	OR017508
24.04.2019	GMS2019-130	#130	Tadpole	Tail clip	40	Samar	32.43455, 34.89416	OR016989	OR017509
24.04.2019	GMS2019-134	#134	Tadpole	Tail clip	40	Samar	32.43455, 34.89416	OR017314	OR017510
28.05.2019	GMS2019-136	#136	Tadpole	Tail clip	35	Ramat Dalton	33.0221,35.4569	OR016988	OR017511
28.05.2019	GMS2019-137	#137	Tadpole	Tail clip	41	Sasa	33.03225, 35.39123	OR016987	OR017512
28.05.2019	GMS2019-138	#138	Tadpole	Tail clip	41	Sasa	33.03225, 35.39123	OR017017	OR017513
28.05.2019	GMS2019-139	#139	Tadpole	Tail clip	41	Sasa	33.03225, 35.39123	OR017016	OR017514
28.05.2019	GMS2019-140	#140	Tadpole	Tail clip	41	Sasa	33.03225, 35.39123	OR017101	OR017515
28.05.2019	GMS2019-141	#141	Tadpole	Tail clip	41	Sasa	33.03225, 35.39123	OR017134	OR017516
28.05.2019	GMS2019-142	#142	Tadpole	Tail clip	41	Sasa	33.03225, 35.39123	OR017053	OR017517
28.05.2019	GMS2019-143	#143	Tadpole	Tail clip	41	Sasa	33.03225, 35.39123	OR017133	OR017518
28.05.2019	GMS2019-144	#144	Tadpole	Tail clip	41	Sasa	33.03225, 35.39123	OR017132	OR017519
28.05.2019	GMS2019-145	#145	Tadpole	Tail clip	41	Sasa	33.03225, 35.39123	OR017077	OR017520
28.05.2019	GMS2019-146	#146	Tadpole	Tail clip	41	Sasa	33.03225, 35.39123	OR017272	OR017521
28.05.2019	GMS2019-148	#148	Tadpole	Tail clip	41	Sasa	33.03225, 35.39123	OR017015	OR017522
28.05.2019	GMS2019-149	#149	Tadpole	Tail clip	41	Sasa	33.03225, 35.39123	OR017076	OR017523
28.05.2019	GMS2019-151	#151	Tadpole	Tail clip	41	Sasa	33.03225, 35.39123	OR017312	OR017525
28.05.2019	GMS2019-152	#152	Tadpole	Tail clip	41	Sasa	33.03225, 35.39123	OR017131	OR017526
28.05.2019	GMS2019-153	#153	Tadpole	Tail clip	41	Sasa	33.03225, 35.39123	OR017014	OR017527
28.05.2019	GMS2019-155	#155	Tadpole	Tail clip	41	Sasa	33.03225, 35.39123	OR017013	OR017528

Date	Sample ID	Individual ID	Life Stage	Type of Sample	Population No.	Pool	Coordinates	GenBank accession number (16S)	GenBank accession number (COI)
28.05.2019	GMS2019-156	#156	Tadpole	Tail clip	41	Sasa	33.03225, 35.39123	OR017012	OR017529
28.05.2019	GMS2019-158	#158	Tadpole	Tail clip	41	Sasa	33.03225, 35.39123	OR017011	OR017530
16.04.2019	GMS2019-160	#160	Adult	Toe clip	29	Maskeret Batia	31.8481,34.8482	OR016986	OR017532
16.04.2019	GMS2019-161	#161	Adult	Toe clip	29	Maskeret Batia	31.8481,34.8482	OR017231	OR017533
16.04.2019	GMS2019-162	#162	Adult	Toe clip	29	Maskeret Batia	31.8481,34.8482	OR017230	OR017534
16.04.2019	GMS2019-163	#163	Adult	Toe clip	29	Maskeret Batia	31.8481,34.8482	OR017296	OR017535
18.03.2019	GMS2019-165	#165	Adult	Toe clip	45	Tel Bezek	32.91367, 35.76155	OR017435	OR017536
18.03.2019	GMS2019-166	#166	Adult	Toe clip	45	Tel Bezek	32.91367, 35.76155	OR017407	OR017537
05.03.2020	GMS2019-170	#170	Tadpole	Tail clip	43	Soreq	31.9347878, 34.7396734	OR017271	OR017538
05.03.2020	GMS2019-172	#172	Tadpole	Tail clip	43	Soreq	31.9347878, 34.7396734	OR017229	OR017539
05.03.2020	GMS2019-173	#173	Tadpole	Tail clip	43	Soreq	31.9347878, 34.7396734	OR017270	OR017540
05.03.2020	GMS2019-174	#174	Tadpole	Tail clip	43	Soreq	31.9347878, 34.7396734	OR017299	OR017541
05.03.2020	GMS2019-175	#175	Tadpole	Tail clip	43	Soreq	31.9347878, 34.7396734	OR017269	OR017542
05.03.2020	GMS2019-176	#176	Tadpole	Tail clip	43	Soreq	31.9347878, 34.7396734	OR016985	OR017543
05.03.2020	GMS2019-177	#177	Tadpole	Tail clip	43	Soreq	31.9347878, 34.7396734	OR017010	OR017544
05.03.2020	GMS2019-178	#178	Tadpole	Tail clip	43	Soreq	31.9347878, 34.7396734	OR017009	OR017545
05.03.2020	GMS2019-179	#179	Tadpole	Tail clip	43	Soreq	31.9347878, 34.7396734	OR017268	OR017546
05.03.2020	GMS2019-180	#180	Tadpole	Tail clip	43	Soreq	31.9347878, 34.7396734	OR017102	OR017547
05.03.2020	GMS2019-181	#181	Tadpole	Tail clip	43	Soreq	31.9347878, 34.7396734	OR017092	OR017548
05.03.2020	GMS2019-182	#182	Tadpole	Tail clip	43	Soreq	31.9347878, 34.7396734	OR017075	OR017549
05.03.2020	GMS2019-183	#183	Tadpole	Tail clip	43	Soreq	31.9347878, 34.7396734	OR017289	OR017550
05.03.2020	GMS2019-184	#184	Tadpole	Tail clip	43	Soreq	31.9347878, 34.7396734	OR017228	OR017551
05.03.2020	GMS2019-186	#186	Tadpole	Tail clip	43	Soreq	31.9347878, 34.7396734	OR016984	OR017552
05.03.2020	GMS2019-187	#187	Tadpole	Tail clip	43	Soreq	31.9347878, 34.7396734	OR017298	OR017553
05.03.2020	GMS2019-189	#189	Tadpole	Tail clip	43	Soreq	31.9347878, 34.7396734	OR017297	OR017554
05.03.2020	GMS2019-190	#190	Tadpole	Tail clip	43	Soreq	31.9347878, 34.7396734	OR017227	OR017555
07.03.2020	GMS2019-192	#192	Tadpole	Tail clip	38	Rehovot	31.90717, 34.8425	OR017311	OR017556

Date	Sample ID	Individual ID	Life Stage	Type of Sample	Population No.	Pool	Coordinates	GenBank accession number (16S)	GenBank accession number (COI)
07.03.2020	GMS2019-193	#193	Tadpole	Tail clip	38	Rehovot	31.90717, 34.8425	OR017267	OR017557
07.03.2020	GMS2019-195	#195	Tadpole	Tail clip	38	Rehovot	31.90717, 34.8425	OR017226	OR017558
07.03.2020	GMS2019-197	#197	Tadpole	Tail clip	38	Rehovot	31.90717, 34.8425	OR017288	OR017559
07.03.2020	GMS2019-198	#198	Tadpole	Tail clip	38	Rehovot	31.90717, 34.8425	OR017319	OR017560
07.03.2020	GMS2019-199	#199	Tadpole	Tail clip	38	Rehovot	31.90717, 34.8425	OR017287	OR017561
07.03.2020	GMS2019-200	#200	Tadpole	Tail clip	38	Rehovot	31.90717, 34.8425	OR017225	OR017562
04.07.2019	GMS2019-203	#203	Adult	Toe clip	47	Zuriman South	33.09659, 35.83913	OR017406	OR017564
04.07.2019	GMS2019-206	#206	Adult	Toe clip	47	Zuriman South	33.09659, 35.83913	OR017405	OR017565
04.07.2019	GMS2019-208	#208	Adult	Toe clip	47	Zuriman South	33.09659, 35.83913	OR017404	OR017566
04.07.2019	GMS2019-209	#209	Adult	Toe clip	47	Zuriman South	33.09659, 35.83913	OR017403	OR017567
04.07.2019	GMS2019-210	#210	Adult	Toe clip	47	Zuriman South	33.09659, 35.83913	OR017402	OR017568
04.07.2019	GMS2019-211	#211	Tadpole	Tail clip	47	Zuriman South	33.09659, 35.83913	OR017440	OR017569
04.07.2019	GMS2019-213	#213	Tadpole	Tail clip	47	Zuriman South	33.09659, 35.83913	OR017418	OR017570
28.05.2019	GMS2019-214	#214	Tadpole	Tail clip	9	Dalton Moshav	33.01621, 35.48064	OR017130	OR017571
28.05.2019	GMS2019-215	#215	Tadpole	Tail clip	9	Dalton Moshav	33.01621, 35.48064	OR017008	OR017572
28.05.2019	GMS2019-217	#217	Tadpole	Tail clip	9	Dalton Moshav	33.01621, 35.48064	OR017007	OR017573
28.05.2019	GMS2019-218	#218	Tadpole	Tail clip	9	Dalton Moshav	33.01621, 35.48064	OR017100	OR017574
28.05.2019	GMS2019-219	#219	Tadpole	Tail clip	9	Dalton Moshav	33.01621, 35.48064	OR017006	OR017575
28.05.2019	GMS2019-220	#220	Tadpole	Tail clip	9	Dalton Moshav	33.01621, 35.48064	OR017005	OR017576
28.05.2019	GMS2019-221	#221	Tadpole	Tail clip	9	Dalton Moshav	33.01621, 35.48064	OR017224	OR017577
28.05.2019	GMS2019-222	#222	Tadpole	Tail clip	9	Dalton Moshav	33.01621, 35.48064	OR017004	OR017578
28.05.2019	GMS2019-223	#223	Tadpole	Tail clip	9	Dalton Moshav	33.01621, 35.48064	OR017223	OR017579
28.05.2019	GMS2019-224	#224	Tadpole	Tail clip	9	Dalton Moshav	33.01621, 35.48064	OR017108	OR017580
28.05.2019	GMS2019-226	#226	Tadpole	Tail clip	9	Dalton Moshav	33.01621, 35.48064	OR017003	OR017581
28.05.2019	GMS2019-227	#227	Tadpole	Tail clip	9	Dalton Moshav	33.01621, 35.48064	OR017002	OR017582
28.05.2019	GMS2019-228	#228	Tadpole	Tail clip	9	Dalton Moshav	33.01621, 35.48064	OR017001	OR017583
28.05.2019	GMS2019-229	#229	Tadpole	Tail clip	9	Dalton Moshav	33.01621, 35.48064	OR017129	OR017584

Date	Sample ID	Individual ID	Life Stage	Type of Sample	Population No.	Pool	Coordinates	GenBank accession number (16S)	GenBank accession number (COI)
28.05.2019	GMS2019-230	#230	Tadpole	Tail clip	9	Dalton Moshav	33.01621, 35.48064	OR017000	OR017585
28.05.2019	GMS2019-231	#231	Tadpole	Tail clip	9	Dalton Moshav	33.01621, 35.48064	OR017107	OR017586
28.05.2019	GMS2019-232	#232	Tadpole	Tail clip	9	Dalton Moshav	33.01621, 35.48064	OR017222	OR017587
28.05.2019	GMS2019-234	#234	Tadpole	Tail clip	9	Dalton Moshav	33.01621, 35.48064	OR016983	OR017588
28.05.2019	GMS2019-236	#236	Tadpole	Tail clip	35	Ramat Dalton	33.0221,35.4569	OR017128	OR017589
28.05.2019	GMS2019-237	#237	Tadpole	Tail clip	35	Ramat Dalton	33.0221,35.4569	OR016999	OR017590
28.05.2019	GMS2019-238	#238	Tadpole	Tail clip	35	Ramat Dalton	33.0221,35.4569	OR016998	OR017591
28.05.2019	GMS2019-239	#239	Tadpole	Tail clip	35	Ramat Dalton	33.0221,35.4569	OR017099	OR017592
28.05.2019	GMS2019-241	#241	Tadpole	Tail clip	35	Ramat Dalton	33.0221,35.4569	OR016982	OR017593
28.05.2019	GMS2019-242	#242	Tadpole	Tail clip	35	Ramat Dalton	33.0221,35.4569	OR017305	OR017594
28.05.2019	GMS2019-243	#243	Tadpole	Tail clip	35	Ramat Dalton	33.0221,35.4569	OR016997	OR017595
28.05.2019	GMS2019-244	#244	Tadpole	Tail clip	35	Ramat Dalton	33.0221,35.4569	OR017127	OR017596
28.05.2019	GMS2019-245	#245	Tadpole	Tail clip	35	Ramat Dalton	33.0221,35.4569	OR017126	OR017597
28.05.2019	GMS2019-246	#246	Tadpole	Tail clip	35	Ramat Dalton	33.0221,35.4569	OR017125	OR017598
28.05.2019	GMS2019-247	#247	Tadpole	Tail clip	35	Ramat Dalton	33.0221,35.4569	OR017098	OR017599
28.05.2019	GMS2019-248	#248	Tadpole	Tail clip	35	Ramat Dalton	33.0221,35.4569	OR017221	OR017600
28.05.2019	GMS2019-249	#249	Tadpole	Tail clip	35	Ramat Dalton	33.0221,35.4569	OR016912	OR017601
28.05.2019	GMS2019-250	#250	Tadpole	Tail clip	35	Ramat Dalton	33.0221,35.4569	OR016789	OR017602
28.05.2019	GMS2019-251	#251	Tadpole	Tail clip	35	Ramat Dalton	33.0221,35.4569	OR017106	OR017603
28.05.2019	GMS2019-252	#252	Tadpole	Tail clip	35	Ramat Dalton	33.0221,35.4569	OR016788	OR017604
28.05.2019	GMS2019-253	#253	Tadpole	Tail clip	35	Ramat Dalton	33.0221,35.4569	OR017124	OR017605
28.05.2019	GMS2019-254	#254	Tadpole	Tail clip	35	Ramat Dalton	33.0221,35.4569	OR016911	OR017606
28.05.2019	GMS2019-255	#255	Tadpole	Tail clip	35	Ramat Dalton	33.0221,35.4569	OR017123	OR017607
07.03.2020	GMS2019-276	#276	Tadpole	Tail clip	38	Rehovot	31.90717, 34.8425	OR017318	OR017608
07.03.2020	GMS2019-277	#277	Tadpole	Tail clip	38	Rehovot	31.90717, 34.8425	OR017317	OR017609
07.03.2020	GMS2019-278	#278	Tadpole	Tail clip	38	Rehovot	31.90717, 34.8425	OR016981	OR017610
07.03.2020	GMS2019-279	#279	Tadpole	Tail clip	38	Rehovot	31.90717, 34.8425	OR017316	OR017611

Date	Sample ID	Individual ID	Life Stage	Type of Sample	Population No.	Pool	Coordinates	GenBank accession number (16S)	GenBank accession number (COI)
07.03.2020	GMS2019-280	#280	Tadpole	Tail clip	38	Rehovot	31.90717, 34.8425	OR017266	OR017612
07.03.2020	GMS2019-281	#281	Tadpole	Tail clip	38	Rehovot	31.90717, 34.8425	OR017286	OR017613
07.03.2020	GMS2019-282	#282	Tadpole	Tail clip	38	Rehovot	31.90717, 34.8425	OR017220	OR017614
07.03.2020	GMS2019-283	#283	Tadpole	Tail clip	38	Rehovot	31.90717, 34.8425	OR017219	OR017615
07.03.2020	GMS2019-284	#284	Tadpole	Tail clip	38	Rehovot	31.90717, 34.8425	OR016980	OR017616
07.03.2020	GMS2019-285	#285	Tadpole	Tail clip	38	Rehovot	31.90717, 34.8425	OR017218	OR017617
07.03.2020	GMS2019-286	#286	Tadpole	Tail clip	38	Rehovot	31.90717, 34.8425	OR017217	OR017618
10.03.2020	GMS2019-289	#289	Tadpole	Tail clip	5	Bareket	32.01338, 34.9644	OR016910	OR017619
10.03.2020	GMS2019-290	#290	Tadpole	Tail clip	5	Bareket	32.01338, 34.9644	OR017146	OR017620
10.03.2020	GMS2019-291	#291	Tadpole	Tail clip	5	Bareket	32.01338, 34.9644	OR017145	OR017621
10.03.2020	GMS2019-292	#292	Tadpole	Tail clip	5	Bareket	32.01338, 34.9644	OR017144	OR017622
10.03.2020	GMS2019-293	#293	Tadpole	Tail clip	5	Bareket	32.01338, 34.9644	OR017143	OR017623
10.03.2020	GMS2019-294	#294	Tadpole	Tail clip	5	Bareket	32.01338, 34.9644	OR016909	OR017624
10.03.2020	GMS2019-295	#295	Tadpole	Tail clip	5	Bareket	32.01338, 34.9644	OR016908	OR017625
10.03.2020	GMS2019-296	#296	Tadpole	Tail clip	5	Bareket	32.01338, 34.9644	OR017142	OR017626
10.03.2020	GMS2019-297	#297	Tadpole	Tail clip	5	Bareket	32.01338, 34.9644	OR017141	OR017627
10.03.2020	GMS2019-298	#298	Tadpole	Tail clip	5	Bareket	32.01338, 34.9644	OR017140	OR017628
10.03.2020	GMS2019-299	#299	Tadpole	Tail clip	5	Bareket	32.01338, 34.9644	OR016907	OR017629
10.03.2020	GMS2019-300	#300	Tadpole	Tail clip	5	Bareket	32.01338, 34.9644	OR016906	OR017630
10.03.2020	GMS2019-301	#301	Tadpole	Tail clip	5	Bareket	32.01338, 34.9644	OR017097	OR017631
10.03.2020	GMS2019-302	#302	Tadpole	Tail clip	5	Bareket	32.01338, 34.9644	OR016905	OR017632
10.03.2020	GMS2019-303	#303	Tadpole	Tail clip	5	Bareket	32.01338, 34.9644	OR016904	OR017633
10.03.2020	GMS2019-306	#306	Tadpole	Tail clip	5	Bareket	32.01338, 34.9644	OR017139	OR017634
10.03.2020	GMS2019-307	#307	Tadpole	Tail clip	15	Elad	32.04396, 34.95554	OR016979	OR017635
10.03.2020	GMS2019-308	#308	Tadpole	Tail clip	15	Elad	32.04396, 34.95554	OR016978	OR017636
10.03.2020	GMS2019-309	#309	Tadpole	Tail clip	15	Elad	32.04396, 34.95554	OR016977	OR017637
10.03.2020	GMS2019-310	#310	Tadpole	Tail clip	15	Elad	32.04396, 34.95554	OR016976	OR017638

Date	Sample ID	Individual ID	Life Stage	Type of Sample	Population No.	Pool	Coordinates	GenBank accession number (16S)	GenBank accession number (COI)
10.03.2020	GMS2019-311	#311	Tadpole	Tail clip	15	Elad	32.04396, 34.95554	OR016975	OR017639
10.03.2020	GMS2019-313	#313	Tadpole	Tail clip	15	Elad	32.04396, 34.95554	OR016974	OR017640
10.03.2020	GMS2019-314	#314	Tadpole	Tail clip	15	Elad	32.04396, 34.95554	OR016903	OR017641
10.03.2020	GMS2019-315	#315	Tadpole	Tail clip	15	Elad	32.04396, 34.95554	OR016973	OR017642
10.03.2020	GMS2019-316	#316	Tadpole	Tail clip	15	Elad	32.04396, 34.95554	OR016972	OR017643
10.03.2020	GMS2019-317	#317	Tadpole	Tail clip	15	Elad	32.04396, 34.95554	OR017284	OR017644
10.03.2020	GMS2019-318	#318	Tadpole	Tail clip	15	Elad	32.04396, 34.95554	OR017283	OR017645
10.03.2020	GMS2019-319	#319	Tadpole	Tail clip	15	Elad	32.04396, 34.95554	OR016971	OR017646
10.03.2020	GMS2019-320	#320	Tadpole	Tail clip	15	Elad	32.04396, 34.95554	OR016970	OR017647
10.03.2020	GMS2019-321	#321	Tadpole	Tail clip	15	Elad	32.04396, 34.95554	OR016969	OR017648
10.03.2020	GMS2019-322	#322	Tadpole	Tail clip	15	Elad	32.04396, 34.95554	OR016968	OR017649
10.03.2020	GMS2019-323	#323	Tadpole	Tail clip	15	Elad	32.04396, 34.95554	OR016967	OR017650
10.03.2020	GMS2019-324	#324	Tadpole	Tail clip	15	Elad	32.04396, 34.95554	OR016966	OR017651
10.03.2020	GMS2019-325	#325	Tadpole	Tail clip	15	Elad	32.04396, 34.95554	OR016965	OR017652
10.03.2020	GMS2019-326	#326	Tadpole	Tail clip	15	Elad	32.04396, 34.95554	OR016964	OR017653
15.03.2020	GMS2019-329	#329	Adult	Toe clip	32	Migdal Zedek	32.07856, 34.95952	OR016963	OR017654
15.03.2020	GMS2019-330	#330	Tadpole	Tail clip	32	Migdal Zedek	32.07856, 34.95952	OR016962	OR017655
15.03.2020	GMS2019-331	#331	Tadpole	Tail clip	32	Migdal Zedek	32.07856, 34.95952	OR016961	OR017656
15.03.2020	GMS2019-332	#332	Tadpole	Tail clip	32	Migdal Zedek	32.07856, 34.95952	OR016960	OR017657
15.03.2020	GMS2019-333	#333	Tadpole	Tail clip	32	Migdal Zedek	32.07856, 34.95952	OR016959	OR017658
15.03.2020	GMS2019-335	#335	Tadpole	Tail clip	32	Migdal Zedek	32.07856, 34.95952	OR016958	OR017659
16.03.2020	GMS2019-336	#336	Tadpole	Tail clip	44	Tel Afeq	32.10573, 34.93342	OR017216	OR017660
16.03.2020	GMS2019-337	#337	Tadpole	Tail clip	44	Tel Afeq	32.10573, 34.93342	OR017138	OR017661
16.03.2020	GMS2019-338	#338	Tadpole	Tail clip	44	Tel Afeq	32.10573, 34.93342	OR016902	OR017662
16.03.2020	GMS2019-340	#340	Tadpole	Tail clip	44	Tel Afeq	32.10573, 34.93342	OR017137	OR017663
16.03.2020	GMS2019-341	#341	Tadpole	Tail clip	44	Tel Afeq	32.10573, 34.93342	OR017215	OR017664
16.03.2020	GMS2019-342	#342	Tadpole	Tail clip	44	Tel Afeq	32.10573, 34.93342	OR017136	OR017665

Date	Sample ID	Individual ID	Life Stage	Type of Sample	Population No.	Pool	Coordinates	GenBank accession number (16S)	GenBank accession number (COI)
16.03.2020	GMS2019-343	#343	Tadpole	Tail clip	44	Tel Afeq	32.10573, 34.93342	OR017135	OR017666
16.03.2020	GMS2019-344	#344	Tadpole	Tail clip	44	Tel Afeq	32.10573, 34.93342	OR017214	OR017667
16.03.2020	GMS2019-345	#345	Tadpole	Tail clip	44	Tel Afeq	32.10573, 34.93342	OR017096	OR017668
16.03.2020	GMS2019-346	#346	Tadpole	Tail clip	44	Tel Afeq	32.10573, 34.93342	OR017095	OR017669
16.03.2020	GMS2019-347	#347	Tadpole	Tail clip	44	Tel Afeq	32.10573, 34.93342	OR017213	OR017670
16.03.2020	GMS2019-348	#348	Tadpole	Tail clip	44	Tel Afeq	32.10573, 34.93342	OR017094	OR017671
16.03.2020	GMS2019-349	#349	Tadpole	Tail clip	44	Tel Afeq	32.10573, 34.93342	OR017212	OR017672
16.03.2020	GMS2019-350	#350	Tadpole	Tail clip	44	Tel Afeq	32.10573, 34.93342	OR016957	OR017673
16.03.2020	GMS2019-351	#351	Tadpole	Tail clip	44	Tel Afeq	32.10573, 34.93342	OR017265	OR017674
16.03.2020	GMS2019-353	#353	Tadpole	Tail clip	44	Tel Afeq	32.10573, 34.93342	OR017211	OR017675
16.03.2020	GMS2019-354	#354	Tadpole	Tail clip	44	Tel Afeq	32.10573, 34.93342	OR017310	OR017676
16.03.2020	GMS2019-355	#355	Tadpole	Tail clip	44	Tel Afeq	32.10573, 34.93342	OR016956	OR017677
16.03.2020	GMS2019-356	#356	Tadpole	Tail clip	21	Hakfar Hayarok	32.13554, 34.81243	OR017295	OR017678
16.03.2020	GMS2019-357	#357	Tadpole	Tail clip	21	Hakfar Hayarok	32.13554, 34.81243	OR016901	OR017679
16.03.2020	GMS2019-358	#358	Tadpole	Tail clip	21	Hakfar Hayarok	32.13554, 34.81243	OR016900	OR017680
16.03.2020	GMS2019-359	#359	Tadpole	Tail clip	21	Hakfar Hayarok	32.13554, 34.81243	OR017264	OR017681
16.03.2020	GMS2019-360	#360	Tadpole	Tail clip	21	Hakfar Hayarok	32.13554, 34.81243	OR017210	OR017682
16.03.2020	GMS2019-361	#361	Tadpole	Tail clip	21	Hakfar Hayarok	32.13554, 34.81243	OR016899	OR017683
16.03.2020	GMS2019-363	#363	Tadpole	Tail clip	21	Hakfar Hayarok	32.13554, 34.81243	OR017263	OR017684
16.03.2020	GMS2019-364	#364	Tadpole	Tail clip	21	Hakfar Hayarok	32.13554, 34.81243	OR016898	OR017685
16.03.2020	GMS2019-365	#365	Tadpole	Tail clip	21	Hakfar Hayarok	32.13554, 34.81243	OR016897	OR017686
16.03.2020	GMS2019-366	#366	Tadpole	Tail clip	21	Hakfar Hayarok	32.13554, 34.81243	OR017262	OR017687
16.03.2020	GMS2019-367	#367	Tadpole	Tail clip	21	Hakfar Hayarok	32.13554, 34.81243	OR017091	OR017688
16.03.2020	GMS2019-368	#368	Tadpole	Tail clip	21	Hakfar Hayarok	32.13554, 34.81243	OR017261	OR017689
16.03.2020	GMS2019-369	#369	Tadpole	Tail clip	21	Hakfar Hayarok	32.13554, 34.81243	OR017090	OR017690
16.03.2020	GMS2019-370	#370	Tadpole	Tail clip	21	Hakfar Hayarok	32.13554, 34.81243	OR017294	OR017691
16.03.2020	GMS2019-371	#371	Tadpole	Tail clip	21	Hakfar Hayarok	32.13554, 34.81243	OR017089	OR017692

Date	Sample ID	Individual ID	Life Stage	Type of Sample	Population No.	Pool	Coordinates	GenBank accession number (16S)	GenBank accession number (COI)
16.03.2020	GMS2019-372	#372	Tadpole	Tail clip	21	Hakfar Hayarok	32.13554, 34.81243	OR017260	OR017693
16.03.2020	GMS2019-373	#373	Tadpole	Tail clip	21	Hakfar Hayarok	32.13554, 34.81243	OR017259	OR017694
16.03.2020	GMS2019-374	#374	Tadpole	Tail clip	21	Hakfar Hayarok	32.13554, 34.81243	OR017258	OR017695
16.03.2020	GMS2019-375	#375	Tadpole	Tail clip	21	Hakfar Hayarok	32.13554, 34.81243	OR017088	OR017696
16.03.2020	GMS2019-378	#378	Tadpole	Tail clip	22	Herzliya Park	32.17216, 34.82457	OR017087	OR017697
16.03.2020	GMS2019-379	#379	Tadpole	Tail clip	22	Herzliya Park	32.17216, 34.82457	OR017209	OR017698
16.03.2020	GMS2019-380	#380	Tadpole	Tail clip	22	Herzliya Park	32.17216, 34.82457	OR016896	OR017699
16.03.2020	GMS2019-381	#381	Tadpole	Tail clip	22	Herzliya Park	32.17216, 34.82457	OR017208	OR017700
16.03.2020	GMS2019-382	#382	Tadpole	Tail clip	22	Herzliya Park	32.17216, 34.82457	OR017207	OR017701
16.03.2020	GMS2019-383	#383	Tadpole	Tail clip	22	Herzliya Park	32.17216, 34.82457	OR017206	OR017702
16.03.2020	GMS2019-384	#384	Tadpole	Tail clip	22	Herzliya Park	32.17216, 34.82457	OR017205	OR017703
16.03.2020	GMS2019-385	#385	Tadpole	Tail clip	22	Herzliya Park	32.17216, 34.82457	OR016895	OR017704
16.03.2020	GMS2019-386	#386	Tadpole	Tail clip	22	Herzliya Park	32.17216, 34.82457	OR016955	OR017705
16.03.2020	GMS2019-387	#387	Tadpole	Tail clip	22	Herzliya Park	32.17216, 34.82457	OR017204	OR017706
12.04.2020	GMS2019-389	#389	Tadpole	Tail clip	19	Gaash North	32.22906, 34.8313	OR017203	OR017707
12.04.2020	GMS2019-390	#390	Tadpole	Tail clip	19	Gaash North	32.22906, 34.8313	OR017073	OR017708
12.04.2020	GMS2019-391	#391	Tadpole	Tail clip	19	Gaash North	32.22906, 34.8313	OR017112	OR017709
12.04.2020	GMS2019-392	#392	Tadpole	Tail clip	19	Gaash North	32.22906, 34.8313	OR017074	OR017710
12.04.2020	GMS2019-393	#393	Tadpole	Tail clip	19	Gaash North	32.22906, 34.8313	OR016793	OR017711
12.04.2020	GMS2019-394	#394	Tadpole	Tail clip	19	Gaash North	32.22906, 34.8313	OR017086	OR017712
12.04.2020	GMS2019-395	#395	Tadpole	Tail clip	19	Gaash North	32.22906, 34.8313	OR017085	OR017713
10.03.2020	GMS2019-397	#397	Tadpole	Tail clip	5	Bareket	32.01338, 34.9644	OR016894	OR017714
10.03.2020	GMS2019-399	#399	Tadpole	Tail clip	5	Bareket	32.01338, 34.9644	OR016893	OR017715
10.03.2020	GMS2019-400	#400	Tadpole	Tail clip	5	Bareket	32.01338, 34.9644	OR016892	OR017716
12.04.2020	GMS2019-401	#401	Tadpole	Tail clip	19	Gaash North	32.22906, 34.8313	OR017202	OR017717
12.04.2020	GMS2019-402	#402	Tadpole	Tail clip	19	Gaash North	32.22906, 34.8313	OR017257	OR017718
12.04.2020	GMS2019-404	#404	Tadpole	Tail clip	19	Gaash North	32.22906, 34.8313	OR017072	OR017719

Date	Sample ID	Individual ID	Life Stage	Type of Sample	Population No.	Pool	Coordinates	GenBank accession number (16S)	GenBank accession number (COI)
12.04.2020	GMS2019-405	#405	Tadpole	Tail clip	19	Gaash North	32.22906, 34.8313	OR017071	OR017720
12.04.2020	GMS2019-406	#406	Tadpole	Tail clip	19	Gaash North	32.22906, 34.8313	OR017201	OR017721
12.04.2020	GMS2019-407	#407	Tadpole	Tail clip	19	Gaash North	32.22906, 34.8313	OR017069	OR017722
12.04.2020	GMS2019-408	#408	Tadpole	Tail clip	1	Agmon Poleg	32.25688, 34.8541	OR016954	OR017723
12.04.2020	GMS2019-409	#409	Tadpole	Tail clip	1	Agmon Poleg	32.25688, 34.8541	OR017256	OR017724
12.04.2020	GMS2019-410	#410	Tadpole	Tail clip	1	Agmon Poleg	32.25688, 34.8541	OR016891	OR017725
12.04.2020	GMS2019-411	#411	Tadpole	Tail clip	1	Agmon Poleg	32.25688, 34.8541	OR016890	OR017726
12.04.2020	GMS2019-412	#412	Tadpole	Tail clip	1	Agmon Poleg	32.25688, 34.8541	OR017200	OR017727
12.04.2020	GMS2019-413	#413	Tadpole	Tail clip	1	Agmon Poleg	32.25688, 34.8541	OR017111	OR017728
12.04.2020	GMS2019-414	#414	Tadpole	Tail clip	1	Agmon Poleg	32.25688, 34.8541	OR016889	OR017729
12.04.2020	GMS2019-415	#415	Tadpole	Tail clip	1	Agmon Poleg	32.25688, 34.8541	OR017255	OR017730
12.04.2020	GMS2019-416	#416	Tadpole	Tail clip	1	Agmon Poleg	32.25688, 34.8541	OR017199	OR017731
12.04.2020	GMS2019-417	#417	Tadpole	Tail clip	1	Agmon Poleg	32.25688, 34.8541	OR016888	OR017732
12.04.2020	GMS2019-418	#418	Tadpole	Tail clip	1	Agmon Poleg	32.25688, 34.8541	OR016953	OR017733
12.04.2020	GMS2019-419	#419	Tadpole	Tail clip	1	Agmon Poleg	32.25688, 34.8541	OR017254	OR017734
12.04.2020	GMS2019-420	#420	Tadpole	Tail clip	1	Agmon Poleg	32.25688, 34.8541	OR017198	OR017735
12.04.2020	GMS2019-421	#421	Tadpole	Tail clip	1	Agmon Poleg	32.25688, 34.8541	OR016952	OR017736
12.04.2020	GMS2019-422	#422	Tadpole	Tail clip	1	Agmon Poleg	32.25688, 34.8541	OR016887	OR017737
12.04.2020	GMS2019-423	#423	Tadpole	Tail clip	1	Agmon Poleg	32.25688, 34.8541	OR017197	OR017738
12.04.2020	GMS2019-426	#426	Tadpole	Tail clip	1	Agmon Poleg	32.25688, 34.8541	OR017253	OR017739
12.04.2020	GMS2019-427	#427	Tadpole	Tail clip	1	Agmon Poleg	32.25688, 34.8541	OR016951	OR017740
12.04.2020	GMS2019-431	#431	Tadpole	Tail clip	39	Ruppin	32.3388, 34.91494	OR017304	OR017741
12.04.2020	GMS2019-434	#434	Tadpole	Tail clip	39	Ruppin	32.3388, 34.91494	OR016950	OR017742
12.04.2020	GMS2019-435	#435	Tadpole	Tail clip	39	Ruppin	32.3388, 34.91494	OR017055	OR017743
12.04.2020	GMS2019-438	#438	Tadpole	Tail clip	39	Ruppin	32.3388, 34.91494	OR017293	OR017744
12.04.2020	GMS2019-440	#440	Tadpole	Tail clip	39	Ruppin	32.3388, 34.91494	OR016886	OR017745
12.04.2020	GMS2019-441	#441	Tadpole	Tail clip	39	Ruppin	32.3388, 34.91494	OR017292	OR017746

Date	Sample ID	Individual ID	Life Stage	Type of Sample	Population No.	Pool	Coordinates	GenBank accession number (16S)	GenBank accession number (COI)
12.04.2020	GMS2019-442	#442	Tadpole	Tail clip	39	Ruppin	32.3388, 34.91494	OR017291	OR017747
12.04.2020	GMS2019-443	#443	Tadpole	Tail clip	39	Ruppin	32.3388, 34.91494	OR016949	OR017748
12.04.2020	GMS2019-444	#444	Tadpole	Tail clip	39	Ruppin	32.3388, 34.91494	OR017252	OR017749
12.04.2020	GMS2019-445	#445	Tadpole	Tail clip	39	Ruppin	32.3388, 34.91494	OR017290	OR017750
12.04.2020	GMS2019-446	#446	Tadpole	Tail clip	39	Ruppin	32.3388, 34.91494	OR016885	OR017751
12.04.2020	GMS2019-448	#448	Tadpole	Tail clip	39	Ruppin	32.3388, 34.91494	OR016948	OR017752
19.09.2020	GMS2019-449	#449	Tadpole	Tail clip	2	Amikam	32.57733, 35.01788	OR017308	OR017753
19.09.2020	GMS2019-450	#450	Tadpole	Tail clip	2	Amikam	32.57733, 35.01788	OR016884	OR017754
19.09.2020	GMS2019-452	#452	Tadpole	Tail clip	2	Amikam	32.57733, 35.01788	OR016883	OR017755
19.09.2020	GMS2019-453	#453	Tadpole	Tail clip	2	Amikam	32.57733, 35.01788	OR017196	OR017756
19.09.2020	GMS2019-454	#454	Tadpole	Tail clip	2	Amikam	32.57733, 35.01788	OR016882	OR017757
19.09.2020	GMS2019-455	#455	Tadpole	Tail clip	2	Amikam	32.57733, 35.01788	OR017122	OR017758
19.09.2020	GMS2019-456	#456	Tadpole	Tail clip	2	Amikam	32.57733, 35.01788	OR017251	OR017759
19.09.2020	GMS2019-457	#457	Tadpole	Tail clip	2	Amikam	32.57733, 35.01788	OR017250	OR017760
19.09.2020	GMS2019-458	#458	Tadpole	Tail clip	2	Amikam	32.57733, 35.01788	OR016947	OR017761
19.09.2020	GMS2019-459	#459	Tadpole	Tail clip	2	Amikam	32.57733, 35.01788	OR017309	OR017762
19.09.2020	GMS2019-460	#460	Tadpole	Tail clip	2	Amikam	32.57733, 35.01788	OR017195	OR017763
19.09.2020	GMS2019-461	#461	Tadpole	Tail clip	2	Amikam	32.57733, 35.01788	OR016881	OR017764
19.09.2020	GMS2019-462	#462	Tadpole	Tail clip	2	Amikam	32.57733, 35.01788	OR016880	OR017765
19.09.2020	GMS2019-463	#463	Tadpole	Tail clip	2	Amikam	32.57733, 35.01788	OR016946	OR017766
19.09.2020	GMS2019-464	#464	Tadpole	Tail clip	2	Amikam	32.57733, 35.01788	OR017068	OR017767
19.09.2020	GMS2019-465	#465	Tadpole	Tail clip	2	Amikam	32.57733, 35.01788	OR017307	OR017768
19.09.2020	GMS2019-466	#466	Tadpole	Tail clip	2	Amikam	32.57733, 35.01788	OR017194	OR017769
19.09.2020	GMS2019-467	#467	Tadpole	Tail clip	2	Amikam	32.57733, 35.01788	OR017193	OR017770
19.09.2020	GMS2019-468	#468	Tadpole	Tail clip	2	Amikam	32.57733, 35.01788	OR016879	OR017771
18.05.2020	GMS2019-469	#469	Tadpole	Tail clip	10	Damon	32.73439, 35.03123	OR016878	OR017772
18.05.2020	GMS2019-470	#470	Tadpole	Tail clip	10	Damon	32.73439, 35.03123	OR016792	OR017773

Date	Sample ID	Individual ID	Life Stage	Type of Sample	Population No.	Pool	Coordinates	GenBank accession number (16S)	GenBank accession number (COI)
18.05.2020	GMS2019-471	#471	Tadpole	Tail clip	10	Damon	32.73439, 35.03123	OR017192	OR017774
18.05.2020	GMS2019-472	#472	Tadpole	Tail clip	10	Damon	32.73439, 35.03123	OR016877	OR017775
18.05.2020	GMS2019-473	#473	Tadpole	Tail clip	10	Damon	32.73439, 35.03123	OR016876	OR017776
18.05.2020	GMS2019-474	#474	Tadpole	Tail clip	10	Damon	32.73439, 35.03123	OR016945	OR017777
18.05.2020	GMS2019-475	#475	Tadpole	Tail clip	10	Damon	32.73439, 35.03123	OR016875	OR017778
18.05.2020	GMS2019-476	#476	Tadpole	Tail clip	10	Damon	32.73439, 35.03123	OR016944	OR017779
18.05.2020	GMS2019-477	#477	Tadpole	Tail clip	10	Damon	32.73439, 35.03123	OR016874	OR017780
18.05.2020	GMS2019-478	#478	Tadpole	Tail clip	10	Damon	32.73439, 35.03123	OR016873	OR017781
18.05.2020	GMS2019-479	#479	Tadpole	Tail clip	10	Damon	32.73439, 35.03123	OR017249	OR017782
18.05.2020	GMS2019-480	#480	Tadpole	Tail clip	10	Damon	32.73439, 35.03123	OR017067	OR017783
18.05.2020	GMS2019-481	#481	Tadpole	Tail clip	10	Damon	32.73439, 35.03123	OR016872	OR017784
18.05.2020	GMS2019-482	#482	Tadpole	Tail clip	10	Damon	32.73439, 35.03123	OR016871	OR017785
18.05.2020	GMS2019-483	#483	Tadpole	Tail clip	10	Damon	32.73439, 35.03123	OR017248	OR017786
18.05.2020	GMS2019-484	#484	Tadpole	Tail clip	10	Damon	32.73439, 35.03123	OR016791	OR017787
18.05.2020	GMS2019-485	#485	Tadpole	Tail clip	10	Damon	32.73439, 35.03123	OR016870	OR017788
18.05.2020	GMS2019-486	#486	Tadpole	Tail clip	10	Damon	32.73439, 35.03123	OR016790	OR017789
18.05.2020	GMS2019-487	#487	Tadpole	Tail clip	10	Damon	32.73439, 35.03123	OR016869	OR017790
18.05.2020	GMS2019-488	#488	Tadpole	Tail clip	10	Damon	32.73439, 35.03123	OR017066	OR017791
21.05.2020	GMS2019-489	#489	Tadpole	Tail clip	7	Bit Keshet	32.72097, 35.40732	OR017103	OR017792
21.05.2020	GMS2019-491	#491	Tadpole	Tail clip	7	Bit Keshet	32.72097, 35.40732	OR016868	OR017793
21.05.2020	GMS2019-493	#493	Tadpole	Tail clip	7	Bit Keshet	32.72097, 35.40732	OR016867	OR017794
21.05.2020	GMS2019-494	#494	Tadpole	Tail clip	7	Bit Keshet	32.72097, 35.40732	OR016866	OR017795
21.05.2020	GMS2019-495	#495	Tadpole	Tail clip	7	Bit Keshet	32.72097, 35.40732	OR016865	OR017796
12.04.2020	GMS2019-498	#498	Tadpole	Tail clip	19	Gaash North	32.22906, 34.8313	OR017070	OR017797
12.04.2020	GMS2019-499	#499	Tadpole	Tail clip	19	Gaash North	32.22906, 34.8313	OR016864	OR017798
01.06.2020	GMS2019-504	#504	Tadpole	Tail clip	30	Menachem	33.07773, 35.2872	OR016863	OR017799
01.06.2020	GMS2019-505	#505	Tadpole	Tail clip	30	Menachem	33.07773, 35.2872	OR016943	OR017800

Date	Sample ID	Individual ID	Life Stage	Type of Sample	Population No.	Pool	Coordinates	GenBank accession number (16S)	GenBank accession number (COI)
01.06.2020	GMS2019-507	#507	Tadpole	Tail clip	30	Menachem	33.07773, 35.2872	OR016862	OR017801
01.06.2020	GMS2019-508	#508	Tadpole	Tail clip	30	Menachem	33.07773, 35.2872	OR016861	OR017802
26.05.2020	GMS2019-509	#509	Tadpole	Tail clip	4	Bajuriyeh	32.86421, 35.78939	OR017401	OR017803
26.05.2020	GMS2019-510	#510	Tadpole	Tail clip	4	Bajuriyeh	32.86421, 35.78939	OR017400	OR017804
26.05.2020	GMS2019-511	#511	Tadpole	Tail clip	4	Bajuriyeh	32.86421, 35.78939	OR017399	OR017805
26.05.2020	GMS2019-512	#512	Tadpole	Tail clip	4	Bajuriyeh	32.86421, 35.78939	OR017398	OR017806
26.05.2020	GMS2019-513	#513	Tadpole	Tail clip	4	Bajuriyeh	32.86421, 35.78939	OR017397	OR017807
26.05.2020	GMS2019-514	#514	Tadpole	Tail clip	4	Bajuriyeh	32.86421, 35.78939	OR017396	OR017808
26.05.2020	GMS2019-515	#515	Tadpole	Tail clip	26	Juchader	32.9300357, 35.8540508	OR017395	OR017809
26.05.2020	GMS2019-516	#516	Tadpole	Tail clip	26	Juchader	32.9300357, 35.8540508	OR017394	OR017810
26.05.2020	GMS2019-517	#517	Tadpole	Tail clip	26	Juchader	32.9300357, 35.8540508	OR017441	OR017811
26.05.2020	GMS2019-520	#520	Tadpole	Tail clip	26	Juchader	32.9300357, 35.8540508	OR017393	OR017812
26.05.2020	GMS2019-523	#523	Tadpole	Tail clip	26	Juchader	32.9300357, 35.8540508	OR017392	OR017813
26.05.2020	GMS2019-524	#524	Tadpole	Tail clip	26	Juchader	32.9300357, 35.8540508	OR017430	OR017814
26.05.2020	GMS2019-540	#540	Tadpole	Tail clip	26	Juchader	32.9300357, 35.8540508	OR017391	OR017815
26.05.2020	GMS2019-541	#541	Tadpole	Tail clip	26	Juchader	32.9300357, 35.8540508	OR017390	OR017816
26.05.2020	GMS2019-542	#542	Tadpole	Tail clip	17	Farej East	32.9611912, 35.8359439	OR017389	OR017817
26.05.2020	GMS2019-543	#543	Tadpole	Tail clip	17	Farej East	32.9611912, 35.8359439	OR017437	OR017818
26.05.2020	GMS2019-544	#544	Tadpole	Tail clip	17	Farej East	32.9611912, 35.8359439	OR017425	OR017819
26.05.2020	GMS2019-546	#546	Tadpole	Tail clip	17	Farej East	32.9611912, 35.8359439	OR017388	OR017820
26.05.2020	GMS2019-547	#547	Tadpole	Tail clip	17	Farej East	32.9611912, 35.8359439	OR017429	OR017821
26.05.2020	GMS2019-548	#548	Tadpole	Tail clip	17	Farej East	32.9611912, 35.8359439	OR017434	OR017822
26.05.2020	GMS2019-549	#549	Tadpole	Tail clip	17	Farej East	32.9611912, 35.8359439	OR017424	OR017823
26.05.2020	GMS2019-550	#550	Tadpole	Tail clip	17	Farej East	32.9611912, 35.8359439	OR017387	OR017824
26.05.2020	GMS2019-551	#551	Tadpole	Tail clip	17	Farej East	32.9611912, 35.8359439	OR017332	OR017825
26.05.2020	GMS2019-552	#552	Tadpole	Tail clip	17	Farej East	32.9611912, 35.8359439	OR017439	OR017826
26.05.2020	GMS2019-553	#553	Tadpole	Tail clip	17	Farej East	32.9611912, 35.8359439	OR017386	OR017827

Date	Sample ID	Individual ID	Life Stage	Type of Sample	Population No.	Pool	Coordinates	GenBank accession number (16S)	GenBank accession number (COI)
26.05.2020	GMS2019-555	#555	Tadpole	Tail clip	17	Farej East	32.9611912, 35.8359439	OR017436	OR017828
26.05.2020	GMS2019-556	#556	Tadpole	Tail clip	17	Farej East	32.9611912, 35.8359439	OR017423	OR017829
26.05.2020	GMS2019-557	#557	Tadpole	Tail clip	17	Farej East	32.9611912, 35.8359439	OR017438	OR017830
26.05.2020	GMS2019-558	#558	Tadpole	Tail clip	17	Farej East	32.9611912, 35.8359439	OR017385	OR017831
26.05.2020	GMS2019-559	#559	Tadpole	Tail clip	17	Farej East	32.9611912, 35.8359439	OR017384	OR017832
26.05.2020	GMS2019-561	#561	Tadpole	Tail clip	17	Farej East	32.9611912, 35.8359439	OR017383	OR017833
26.05.2020	GMS2019-563	#563	Tadpole	Tail clip	17	Farej East	32.9611912, 35.8359439	OR017433	OR017834
26.05.2020	GMS2019-567	#567	Tadpole	Tail clip	24	Hushniya	32.99485, 35.81165	OR017382	OR017835
26.05.2020	GMS2019-569	#569	Tadpole	Tail clip	24	Hushniya	32.99485, 35.81165	OR017381	OR017836
26.05.2020	GMS2019-570	#570	Tadpole	Tail clip	24	Hushniya	32.99485, 35.81165	OR017380	OR017837
26.05.2020	GMS2019-571	#571	Tadpole	Tail clip	24	Hushniya	32.99485, 35.81165	OR017379	OR017838
26.05.2020	GMS2019-572	#572	Tadpole	Tail clip	24	Hushniya	32.99485, 35.81165	OR017378	OR017839
26.05.2020	GMS2019-573	#573	Tadpole	Tail clip	24	Hushniya	32.99485, 35.81165	OR017417	OR017840
26.05.2020	GMS2019-574	#574	Tadpole	Tail clip	24	Hushniya	32.99485, 35.81165	OR017377	OR017841
26.05.2020	GMS2019-575	#575	Tadpole	Tail clip	24	Hushniya	32.99485, 35.81165	OR017376	OR017842
26.05.2020	GMS2019-576	#576	Tadpole	Tail clip	24	Hushniya	32.99485, 35.81165	OR017431	OR017843
26.05.2020	GMS2019-577	#577	Tadpole	Tail clip	24	Hushniya	32.99485, 35.81165	OR017375	OR017844
26.05.2020	GMS2019-578	#578	Tadpole	Tail clip	24	Hushniya	32.99485, 35.81165	OR017374	OR017845
26.05.2020	GMS2019-579	#579	Tadpole	Tail clip	24	Hushniya	32.99485, 35.81165	OR017373	OR017846
26.05.2020	GMS2019-581	#581	Tadpole	Tail clip	24	Hushniya	32.99485, 35.81165	OR017372	OR017847
26.05.2020	GMS2019-584	#584	Tadpole	Tail clip	24	Hushniya	32.99485, 35.81165	OR017371	OR017848
26.05.2020	GMS2019-585	#585	Tadpole	Tail clip	24	Hushniya	32.99485, 35.81165	OR017370	OR017849
26.05.2020	GMS2019-586	#586	Tadpole	Tail clip	24	Hushniya	32.99485, 35.81165	OR017334	OR017850
26.05.2020	GMS2019-587	#587	Tadpole	Tail clip	24	Hushniya	32.99485, 35.81165	OR017369	OR017851
26.05.2020	GMS2019-588	#588	Tadpole	Tail clip	24	Hushniya	32.99485, 35.81165	OR017416	OR017852
26.05.2020	GMS2019-592	#592	Tadpole	Tail clip	14	Ein Zivan NT	33.1001028, 35.8094936	OR017368	OR017853
26.05.2020	GMS2019-593	#593	Tadpole	Tail clip	14	Ein Zivan NT	33.1001028, 35.8094936	OR017443	OR017854

Date	Sample ID	Individual ID	Life Stage	Type of Sample	Population No.	Pool	Coordinates	GenBank accession number (16S)	GenBank accession number (COI)
26.05.2020	GMS2019-594	#594	Tadpole	Tail clip	14	Ein Zivan NT	33.1001028, 35.8094936	OR017367	OR017855
26.05.2020	GMS2019-595	#595	Tadpole	Tail clip	14	Ein Zivan NT	33.1001028, 35.8094936	OR017366	OR017856
26.05.2020	GMS2019-596	#596	Tadpole	Tail clip	14	Ein Zivan NT	33.1001028, 35.8094936	OR017365	OR017857
01.06.2020	GMS2019-599	#599	Tadpole	Tail clip	30	Menachem	33.07773, 35.2872	OR016860	OR017858
01.06.2020	GMS2019-600	#600	Tadpole	Tail clip	30	Menachem	33.07773, 35.2872	OR017191	OR017859
26.05.2020	GMS2019-601	#601	Tadpole	Tail clip	14	Ein Zivan NT	33.1001028, 35.8094936	OR017442	OR017860
26.05.2020	GMS2019-602	#602	Tadpole	Tail clip	14	Ein Zivan NT	33.1001028, 35.8094936	OR017364	OR017861
26.05.2020	GMS2019-603	#603	Tadpole	Tail clip	14	Ein Zivan NT	33.1001028, 35.8094936	OR017427	OR017862
26.05.2020	GMS2019-604	#604	Tadpole	Tail clip	14	Ein Zivan NT	33.1001028, 35.8094936	OR017428	OR017863
26.05.2020	GMS2019-605	#605	Tadpole	Tail clip	14	Ein Zivan NT	33.1001028, 35.8094936	OR017363	OR017864
26.05.2020	GMS2019-606	#606	Tadpole	Tail clip	14	Ein Zivan NT	33.1001028, 35.8094936	OR017362	OR017865
26.05.2020	GMS2019-607	#607	Tadpole	Tail clip	14	Ein Zivan NT	33.1001028, 35.8094936	OR017361	OR017866
26.05.2020	GMS2019-608	#608	Tadpole	Tail clip	14	Ein Zivan NT	33.1001028, 35.8094936	OR017360	OR017867
26.05.2020	GMS2019-609	#609	Tadpole	Tail clip	14	Ein Zivan NT	33.1001028, 35.8094936	OR017359	OR017868
26.05.2020	GMS2019-610	#610	Tadpole	Tail clip	14	Ein Zivan NT	33.1001028, 35.8094936	OR017426	OR017869
26.05.2020	GMS2019-611	#611	Tadpole	Tail clip	14	Ein Zivan NT	33.1001028, 35.8094936	OR017358	OR017870
26.05.2020	GMS2019-612	#612	Tadpole	Tail clip	14	Ein Zivan NT	33.1001028, 35.8094936	OR017422	OR017871
26.05.2020	GMS2019-613	#613	Tadpole	Tail clip	14	Ein Zivan NT	33.1001028, 35.8094936	OR017357	OR017872
26.05.2020	GMS2019-614	#614	Tadpole	Tail clip	14	Ein Zivan NT	33.1001028, 35.8094936	OR017356	OR017873
26.05.2020	GMS2019-617	#617	Tadpole	Tail clip	14	Ein Zivan NT	33.1001028, 35.8094936	OR017355	OR017874
01.06.2020	GMS2019-632	#632	Tadpole	Tail clip	30	Menachem	33.07773, 35.2872	OR016859	OR017875
01.06.2020	GMS2019-633	#633	Tadpole	Tail clip	30	Menachem	33.07773, 35.2872	OR016796	OR017876
01.06.2020	GMS2019-634	#634	Tadpole	Tail clip	30	Menachem	33.07773, 35.2872	OR017121	OR017877
01.06.2020	GMS2019-635	#635	Tadpole	Tail clip	30	Menachem	33.07773, 35.2872	OR016858	OR017878
01.06.2020	GMS2019-636	#636	Tadpole	Tail clip	30	Menachem	33.07773, 35.2872	OR016857	OR017879
01.06.2020	GMS2019-637	#637	Tadpole	Tail clip	30	Menachem	33.07773, 35.2872	OR017190	OR017880
01.06.2020	GMS2019-638	#638	Tadpole	Tail clip	30	Menachem	33.07773, 35.2872	OR016942	OR017881

Date	Sample ID	Individual ID	Life Stage	Type of Sample	Population No.	Pool	Coordinates	GenBank accession number (16S)	GenBank accession number (COI)
01.06.2020	GMS2019-639	#639	Tadpole	Tail clip	30	Menachem	33.07773, 35.2872	OR016941	OR017882
01.06.2020	GMS2019-640	#640	Tadpole	Tail clip	30	Menachem	33.07773, 35.2872	OR017189	OR017883
01.06.2020	GMS2019-641	#641	Tadpole	Tail clip	30	Menachem	33.07773, 35.2872	OR017188	OR017884
01.06.2020	GMS2019-644	#644	Tadpole	Tail clip	30	Menachem	33.07773, 35.2872	OR016856	OR017885
01.06.2020	GMS2019-646	#646	Tadpole	Tail clip	30	Menachem	33.07773, 35.2872	OR017065	OR017886
01.06.2020	GMS2019-648	#648	Tadpole	Tail clip	30	Menachem	33.07773, 35.2872	OR017120	OR017887
01.06.2020	GMS2019-649	#649	Tadpole	Tail clip	18	Fassuta	33.0457917, 35.298968	OR017064	OR017888
01.06.2020	GMS2019-650	#650	Tadpole	Tail clip	18	Fassuta	33.0457917, 35.298968	OR017119	OR017889
01.06.2020	GMS2019-651	#651	Tadpole	Tail clip	18	Fassuta	33.0457917, 35.298968	OR017187	OR017890
01.06.2020	GMS2019-652	#652	Tadpole	Tail clip	18	Fassuta	33.0457917, 35.298968	OR017059	OR017891
01.06.2020	GMS2019-653	#653	Tadpole	Tail clip	18	Fassuta	33.0457917, 35.298968	OR017118	OR017892
01.06.2020	GMS2019-654	#654	Tadpole	Tail clip	18	Fassuta	33.0457917, 35.298968	OR017063	OR017893
01.06.2020	GMS2019-655	#655	Tadpole	Tail clip	18	Fassuta	33.0457917, 35.298968	OR017186	OR017894
01.06.2020	GMS2019-656	#656	Tadpole	Tail clip	18	Fassuta	33.0457917, 35.298968	OR017247	OR017895
01.06.2020	GMS2019-657	#657	Tadpole	Tail clip	18	Fassuta	33.0457917, 35.298968	OR017185	OR017896
01.06.2020	GMS2019-658	#658	Tadpole	Tail clip	18	Fassuta	33.0457917, 35.298968	OR017062	OR017897
01.06.2020	GMS2019-659	#659	Tadpole	Tail clip	18	Fassuta	33.0457917, 35.298968	OR017184	OR017898
01.06.2020	GMS2019-660	#660	Tadpole	Tail clip	18	Fassuta	33.0457917, 35.298968	OR017183	OR017899
01.06.2020	GMS2019-661	#661	Tadpole	Tail clip	18	Fassuta	33.0457917, 35.298968	OR017061	OR017900
01.06.2020	GMS2019-662	#662	Tadpole	Tail clip	18	Fassuta	33.0457917, 35.298968	OR017182	OR017901
01.06.2020	GMS2019-663	#663	Tadpole	Tail clip	18	Fassuta	33.0457917, 35.298968	OR017246	OR017902
01.06.2020	GMS2019-664	#664	Tadpole	Tail clip	18	Fassuta	33.0457917, 35.298968	OR017117	OR017903
01.06.2020	GMS2019-665	#665	Tadpole	Tail clip	18	Fassuta	33.0457917, 35.298968	OR017181	OR017904
01.06.2020	GMS2019-666	#666	Tadpole	Tail clip	18	Fassuta	33.0457917, 35.298968	OR017060	OR017905
01.06.2020	GMS2019-667	#667	Tadpole	Tail clip	18	Fassuta	33.0457917, 35.298968	OR017058	OR017906
01.06.2020	GMS2019-671	#671	Tadpole	Tail clip	18	Fassuta	33.0457917, 35.298968	OR017057	OR017907
09.06.2020	GMS2019-674	#674	Tadpole	Tail clip	27	Kash	33.03026, 35.49078	OR016855	OR017908

Date	Sample ID	Individual ID	Life Stage	Type of Sample	Population No.	Pool	Coordinates	GenBank accession number (16S)	GenBank accession number (COI)
09.06.2020	GMS2019-678	#678	Tadpole	Tail clip	27	Kash	33.03026, 35.49078	OR016854	OR017909
09.06.2020	GMS2019-683	#683	Tadpole	Tail clip	27	Kash	33.03026, 35.49078	OR016940	OR017910
09.06.2020	GMS2019-684	#684	Tadpole	Tail clip	27	Kash	33.03026, 35.49078	OR017054	OR017911
09.06.2020	GMS2019-685	#685	Tadpole	Tail clip	27	Kash	33.03026, 35.49078	OR017180	OR017912
09.06.2020	GMS2019-686	#686	Tadpole	Tail clip	27	Kash	33.03026, 35.49078	OR016853	OR017913
09.06.2020	GMS2019-688	#688	Tadpole	Tail clip	27	Kash	33.03026, 35.49078	OR017306	OR017914
09.06.2020	GMS2019-689	#689	Tadpole	Tail clip	27	Kash	33.03026, 35.49078	OR016852	OR017915
09.06.2020	GMS2019-691	#691	Tadpole	Tail clip	27	Kash	33.03026, 35.49078	OR016851	OR017916
09.06.2020	GMS2019-692	#692	Tadpole	Tail clip	12	Dovev	33.05132, 35.41611	OR017116	OR017917
09.06.2020	GMS2019-693	#693	Tadpole	Tail clip	12	Dovev	33.05132, 35.41611	OR016850	OR017918
09.06.2020	GMS2019-694	#694	Tadpole	Tail clip	12	Dovev	33.05132, 35.41611	OR016849	OR017919
09.06.2020	GMS2019-695	#695	Tadpole	Tail clip	12	Dovev	33.05132, 35.41611	OR016939	OR017920
09.06.2020	GMS2019-696	#696	Tadpole	Tail clip	12	Dovev	33.05132, 35.41611	OR016848	OR017921
09.06.2020	GMS2019-697	#697	Tadpole	Tail clip	12	Dovev	33.05132, 35.41611	OR016847	OR017922
09.06.2020	GMS2019-698	#698	Tadpole	Tail clip	12	Dovev	33.05132, 35.41611	OR017245	OR017923
09.06.2020	GMS2019-699	#699	Tadpole	Tail clip	12	Dovev	33.05132, 35.41611	OR016938	OR017924
09.06.2020	GMS2019-700	#700	Tadpole	Tail clip	12	Dovev	33.05132, 35.41611	OR017244	OR017925
09.06.2020	GMS2019-701	#701	Tadpole	Tail clip	12	Dovev	33.05132, 35.41611	OR017243	OR017926
09.06.2020	GMS2019-702	#702	Tadpole	Tail clip	12	Dovev	33.05132, 35.41611	OR017242	OR017927
09.06.2020	GMS2019-703	#703	Tadpole	Tail clip	12	Dovev	33.05132, 35.41611	OR016937	OR017928
09.06.2020	GMS2019-704	#704	Tadpole	Tail clip	12	Dovev	33.05132, 35.41611	OR016936	OR017929
09.06.2020	GMS2019-705	#705	Tadpole	Tail clip	12	Dovev	33.05132, 35.41611	OR017052	OR017930
09.06.2020	GMS2019-706	#706	Tadpole	Tail clip	12	Dovev	33.05132, 35.41611	OR016935	OR017931
09.06.2020	GMS2019-707	#707	Tadpole	Tail clip	12	Dovev	33.05132, 35.41611	OR017241	OR017932
09.06.2020	GMS2019-708	#708	Tadpole	Tail clip	12	Dovev	33.05132, 35.41611	OR016846	OR017933
09.06.2020	GMS2019-709	#709	Tadpole	Tail clip	12	Dovev	33.05132, 35.41611	OR017240	OR017934
09.06.2020	GMS2019-710	#710	Tadpole	Tail clip	12	Dovev	33.05132, 35.41611	OR016934	OR017935

Date	Sample ID	Individual ID	Life Stage	Type of Sample	Population No.	Pool	Coordinates	GenBank accession number (16S)	GenBank accession number (COI)
09.06.2020	GMS2019-711	#711	Tadpole	Tail clip	12	Dovev	33.05132, 35.41611	OR016933	OR017936
09.06.2020	GMS2019-717	#717	Adult	Toe clip	31	Meron	32.9907195, 35.4109757	OR016932	OR017937
09.06.2020	GMS2019-718	#718	Tadpole	Tail clip	31	Meron	32.9907195, 35.4109757	OR016845	OR017938
09.06.2020	GMS2019-719	#719	Tadpole	Tail clip	31	Meron	32.9907195, 35.4109757	OR017301	OR017939
09.06.2020	GMS2019-720	#720	Tadpole	Tail clip	31	Meron	32.9907195, 35.4109757	OR017179	OR017940
09.06.2020	GMS2019-721	#721	Tadpole	Tail clip	31	Meron	32.9907195, 35.4109757	OR017178	OR017941
09.06.2020	GMS2019-722	#722	Tadpole	Tail clip	31	Meron	32.9907195, 35.4109757	OR017177	OR017942
09.06.2020	GMS2019-723	#723	Adult	Toe clip	31	Meron	32.9907195, 35.4109757	OR017176	OR017943
16.06.2020	GMS2019-724	#724	Tadpole	Tail clip	16	Elrom	33.1957953, 35.6757077	OR017415	OR017944
09.06.2020	GMS2019-725	#725	Tadpole	Tail clip	31	Meron	32.9907195, 35.4109757	OR017175	OR017945
09.06.2020	GMS2019-726	#726	Tadpole	Tail clip	42	Shufanim	32.96371, 35.39644	OR016795	OR017946
09.06.2020	GMS2019-727	#727	Tadpole	Tail clip	42	Shufanim	32.96371, 35.39644	OR017239	OR017947
09.06.2020	GMS2019-728	#728	Tadpole	Tail clip	42	Shufanim	32.96371, 35.39644	OR016931	OR017948
09.06.2020	GMS2019-729	#729	Tadpole	Tail clip	42	Shufanim	32.96371, 35.39644	OR017115	OR017949
09.06.2020	GMS2019-730	#730	Tadpole	Tail clip	42	Shufanim	32.96371, 35.39644	OR016844	OR017950
09.06.2020	GMS2019-731	#731	Tadpole	Tail clip	42	Shufanim	32.96371, 35.39644	OR017174	OR017951
09.06.2020	GMS2019-732	#732	Tadpole	Tail clip	42	Shufanim	32.96371, 35.39644	OR017051	OR017952
09.06.2020	GMS2019-733	#733	Tadpole	Tail clip	42	Shufanim	32.96371, 35.39644	OR016843	OR017953
09.06.2020	GMS2019-734	#734	Tadpole	Tail clip	42	Shufanim	32.96371, 35.39644	OR016842	OR017954
09.06.2020	GMS2019-735	#735	Tadpole	Tail clip	42	Shufanim	32.96371, 35.39644	OR016794	OR017955
09.06.2020	GMS2019-736	#736	Tadpole	Tail clip	42	Shufanim	32.96371, 35.39644	OR016841	OR017956
09.06.2020	GMS2019-737	#737	Tadpole	Tail clip	42	Shufanim	32.96371, 35.39644	OR016840	OR017957
09.06.2020	GMS2019-738	#738	Tadpole	Tail clip	42	Shufanim	32.96371, 35.39644	OR017173	OR017958
09.06.2020	GMS2019-739	#739	Tadpole	Tail clip	42	Shufanim	32.96371, 35.39644	OR016839	OR017959
09.06.2020	GMS2019-740	#740	Tadpole	Tail clip	42	Shufanim	32.96371, 35.39644	OR016930	OR017960
09.06.2020	GMS2019-741	#741	Tadpole	Tail clip	42	Shufanim	32.96371, 35.39644	OR016838	OR017961
09.06.2020	GMS2019-742	#742	Tadpole	Tail clip	42	Shufanim	32.96371, 35.39644	OR016837	OR017962

Date	Sample ID	Individual ID	Life Stage	Type of Sample	Population No.	Pool	Coordinates	GenBank accession number (16S)	GenBank accession number (COI)
16.06.2020	GMS2019-743	#743	Tadpole	Tail clip	16	Elrom	33.1957953, 35.6757077	OR017331	OR017963
16.06.2020	GMS2019-744	#744	Tadpole	Tail clip	16	Elrom	33.1957953, 35.6757077	OR017330	OR017964
16.06.2020	GMS2019-746	#746	Tadpole	Tail clip	16	Elrom	33.1957953, 35.6757077	OR017354	OR017965
16.06.2020	GMS2019-747	#747	Tadpole	Tail clip	16	Elrom	33.1957953, 35.6757077	OR017329	OR017966
16.06.2020	GMS2019-748	#748	Tadpole	Tail clip	16	Elrom	33.1957953, 35.6757077	OR017353	OR017967
16.06.2020	GMS2019-749	#749	Tadpole	Tail clip	16	Elrom	33.1957953, 35.6757077	OR017414	OR017968
16.06.2020	GMS2019-750	#750	Tadpole	Tail clip	16	Elrom	33.1957953, 35.6757077	OR017328	OR017969
16.06.2020	GMS2019-751	#751	Tadpole	Tail clip	16	Elrom	33.1957953, 35.6757077	OR017327	OR017970
16.06.2020	GMS2019-752	#752	Tadpole	Tail clip	16	Elrom	33.1957953, 35.6757077	OR017326	OR017971
16.06.2020	GMS2019-753	#753	Tadpole	Tail clip	16	Elrom	33.1957953, 35.6757077	OR017421	OR017972
16.06.2020	GMS2019-754	#754	Tadpole	Tail clip	16	Elrom	33.1957953, 35.6757077	OR017413	OR017973
16.06.2020	GMS2019-755	#755	Tadpole	Tail clip	16	Elrom	33.1957953, 35.6757077	OR017325	OR017974
16.06.2020	GMS2019-757	#757	Tadpole	Tail clip	16	Elrom	33.1957953, 35.6757077	OR017324	OR017975
16.06.2020	GMS2019-758	#758	Tadpole	Tail clip	16	Elrom	33.1957953, 35.6757077	OR017412	OR017976
16.06.2020	GMS2019-759	#759	Tadpole	Tail clip	16	Elrom	33.1957953, 35.6757077	OR017352	OR017977
16.06.2020	GMS2019-760	#760	Tadpole	Tail clip	16	Elrom	33.1957953, 35.6757077	OR017411	OR017978
16.06.2020	GMS2019-764	#764	Tadpole	Tail clip	16	Elrom	33.1957953, 35.6757077	OR017410	OR017979
16.06.2020	GMS2019-765	#765	Tadpole	Tail clip	16	Elrom	33.1957953, 35.6757077	OR017323	OR017980
03.-06.2012	GMS2019-776	#776	Tadpole	Tail clip	8	Canada Park	31.841621,34.993653	OR016836	OR017981
03.-06.2012	GMS2019-777	#777	Tadpole	Tail clip	8	Canada Park	31.841621,34.993653	OR016835	OR017982
03.-06.2012	GMS2019-778	#778	Tadpole	Tail clip	8	Canada Park	31.841621,34.993653	OR016834	OR017983
03.-06.2012	GMS2019-779	#779	Tadpole	Tail clip	8	Canada Park	31.841621,34.993653	OR016833	OR017984
03.-06.2012	GMS2019-780	#780	Tadpole	Tail clip	8	Canada Park	31.841621,34.993653	OR016832	OR017985
03.-06.2012	GMS2019-781	#781	Tadpole	Tail clip	8	Canada Park	31.841621,34.993653	OR016831	OR017986
03.-06.2012	GMS2019-782	#782	Tadpole	Tail clip	8	Canada Park	31.841621,34.993653	OR016830	OR017987
03.-06.2012	GMS2019-783	#783	Tadpole	Tail clip	8	Canada Park	31.841621,34.993653	OR016829	OR017988
03.-06.2012	GMS2019-784	#784	Tadpole	Tail clip	8	Canada Park	31.841621,34.993653	OR016828	OR017989

Date	Sample ID	Individual ID	Life Stage	Type of Sample	Population No.	Pool	Coordinates	GenBank accession number (16S)	GenBank accession number (COI)
03.-06.2012	GMS2019-785	#785	Tadpole	Tail clip	8	Canada Park	31.841621,34.993653	OR016827	OR017990
03.-06.2012	GMS2019-786	#786	Tadpole	Tail clip	8	Canada Park	31.841621,34.993653	OR016826	OR017991
03.-06.2012	GMS2019-787	#787	Tadpole	Tail clip	8	Canada Park	31.841621,34.993653	OR016825	OR017992
03.-06.2012	GMS2019-788	#788	Tadpole	Tail clip	8	Canada Park	31.841621,34.993653	OR016824	OR017993
03.-06.2012	GMS2019-789	#789	Tadpole	Tail clip	8	Canada Park	31.841621,34.993653	OR016823	OR017994
03.-06.2012	GMS2019-790	#790	Tadpole	Tail clip	8	Canada Park	31.841621,34.993653	OR016822	OR017995
03.-06.2012	GMS2019-792	#792	Tadpole	Tail clip	8	Canada Park	31.841621,34.993653	OR016821	OR017996
03.-06.2012	GMS2019-793	#793	Tadpole	Tail clip	8	Canada Park	31.841621,34.993653	OR016820	OR017997
03.-06.2012	GMS2019-794	#794	Tadpole	Tail clip	8	Canada Park	31.841621,34.993653	OR016819	OR017998
03.-06.2012	GMS2019-795	#795	Tadpole	Tail clip	8	Canada Park	31.841621,34.993653	OR016818	OR017999
15.04.2012	GMS2019-796	#796	Tadpole	Tail clip	6	Barkan	32.109511, 35.106012	OR016929	OR018000
15.04.2012	GMS2019-797	#797	Tadpole	Tail clip	6	Barkan	32.109511, 35.106012	OR016928	OR018001
15.04.2012	GMS2019-798	#798	Tadpole	Tail clip	6	Barkan	32.109511, 35.106012	OR016817	OR018002
15.04.2012	GMS2019-799	#799	Tadpole	Tail clip	6	Barkan	32.109511, 35.106012	OR016927	OR018003
15.04.2012	GMS2019-800	#800	Tadpole	Tail clip	6	Barkan	32.109511, 35.106012	OR016926	OR018004
15.04.2012	GMS2019-801	#801	Tadpole	Tail clip	6	Barkan	32.109511, 35.106012	OR016925	OR018005
15.04.2012	GMS2019-802	#802	Tadpole	Tail clip	6	Barkan	32.109511, 35.106012	OR016924	OR018006
15.04.2012	GMS2019-803	#803	Tadpole	Tail clip	6	Barkan	32.109511, 35.106012	OR016816	OR018007
15.04.2012	GMS2019-804	#804	Tadpole	Tail clip	6	Barkan	32.109511, 35.106012	OR016815	OR018008
15.04.2012	GMS2019-805	#805	Tadpole	Tail clip	6	Barkan	32.109511, 35.106012	OR016814	OR018009
15.04.2012	GMS2019-806	#806	Tadpole	Tail clip	6	Barkan	32.109511, 35.106012	OR016813	OR018010
15.04.2012	GMS2019-807	#807	Tadpole	Tail clip	6	Barkan	32.109511, 35.106012	OR016923	OR018011
15.04.2012	GMS2019-808	#808	Tadpole	Tail clip	6	Barkan	32.109511, 35.106012	OR016922	OR018012
15.04.2012	GMS2019-809	#809	Tadpole	Tail clip	6	Barkan	32.109511, 35.106012	OR016921	OR018013
15.04.2012	GMS2019-810	#810	Tadpole	Tail clip	6	Barkan	32.109511, 35.106012	OR016812	OR018014
15.04.2012	GMS2019-811	#811	Tadpole	Tail clip	6	Barkan	32.109511, 35.106012	OR016920	OR018015
15.04.2012	GMS2019-812	#812	Tadpole	Tail clip	6	Barkan	32.109511, 35.106012	OR016919	OR018016

Date	Sample ID	Individual ID	Life Stage	Type of Sample	Population No.	Pool	Coordinates	GenBank accession number (16S)	GenBank accession number (COI)
15.04.2012	GMS2019-813	#813	Tadpole	Tail clip	6	Barkan	32.109511, 35.106012	OR016811	OR018017
15.04.2012	GMS2019-814	#814	Tadpole	Tail clip	6	Barkan	32.109511, 35.106012	OR016810	OR018018
15.04.2012	GMS2019-815	#815	Tadpole	Tail clip	6	Barkan	32.109511, 35.106012	OR016918	OR018019
12.05.2012	GMS2019-841	#841	Tadpole	Tail clip	36	Razania	33.02672, 35.75936	OR017351	OR018020
12.05.2012	GMS2019-842	#842	Tadpole	Tail clip	36	Razania	33.02672, 35.75936	OR017350	OR018021
12.05.2012	GMS2019-843	#843	Tadpole	Tail clip	36	Razania	33.02672, 35.75936	OR017349	OR018022
12.05.2012	GMS2019-844	#844	Tadpole	Tail clip	36	Razania	33.02672, 35.75936	OR017333	OR018023
12.05.2012	GMS2019-845	#845	Tadpole	Tail clip	36	Razania	33.02672, 35.75936	OR017348	OR018024
12.05.2012	GMS2019-846	#846	Tadpole	Tail clip	36	Razania	33.02672, 35.75936	OR017347	OR018025
12.05.2012	GMS2019-847	#847	Tadpole	Tail clip	36	Razania	33.02672, 35.75936	OR017346	OR018026
12.05.2012	GMS2019-848	#848	Tadpole	Tail clip	36	Razania	33.02672, 35.75936	OR017345	OR018027
12.05.2012	GMS2019-849	#849	Tadpole	Tail clip	36	Razania	33.02672, 35.75936	OR017344	OR018028
12.05.2012	GMS2019-850	#850	Tadpole	Tail clip	36	Razania	33.02672, 35.75936	OR017343	OR018029
12.05.2012	GMS2019-851	#851	Tadpole	Tail clip	36	Razania	33.02672, 35.75936	OR017342	OR018030
12.05.2012	GMS2019-852	#852	Tadpole	Tail clip	36	Razania	33.02672, 35.75936	OR017341	OR018031
12.05.2012	GMS2019-853	#853	Tadpole	Tail clip	36	Razania	33.02672, 35.75936	OR017340	OR018032
12.05.2012	GMS2019-854	#854	Tadpole	Tail clip	36	Razania	33.02672, 35.75936	OR017339	OR018033
12.05.2012	GMS2019-855	#855	Tadpole	Tail clip	36	Razania	33.02672, 35.75936	OR017420	OR018034
12.05.2012	GMS2019-856	#856	Tadpole	Tail clip	36	Razania	33.02672, 35.75936	OR017338	OR018035
12.05.2012	GMS2019-857	#857	Tadpole	Tail clip	36	Razania	33.02672, 35.75936	OR017147	OR018036
12.05.2012	GMS2019-858	#858	Tadpole	Tail clip	36	Razania	33.02672, 35.75936	OR017337	OR018037
12.05.2012	GMS2019-859	#859	Tadpole	Tail clip	36	Razania	33.02672, 35.75936	OR017336	OR018038
12.05.2012	GMS2019-860	#860	Tadpole	Tail clip	36	Razania	33.02672, 35.75936	OR017335	OR018039
27.03.2012	GMS2019-861	#861	Tadpole	Tail clip	25	Imam Ali	32.241079, 35.169876	OR017282	OR018040
27.03.2012	GMS2019-862	#862	Tadpole	Tail clip	25	Imam Ali	32.241079, 35.169876	OR017281	OR018041
27.03.2012	GMS2019-863	#863	Tadpole	Tail clip	25	Imam Ali	32.241079, 35.169876	OR017172	OR018042
27.03.2012	GMS2019-864	#864	Tadpole	Tail clip	25	Imam Ali	32.241079, 35.169876	OR017280	OR018043

Date	Sample ID	Individual ID	Life Stage	Type of Sample	Population No.	Pool	Coordinates	GenBank accession number (16S)	GenBank accession number (COI)
27.03.2012	GMS2019-865	#865	Tadpole	Tail clip	25	Imam Ali	32.241079, 35.169876	OR016917	OR018044
27.03.2012	GMS2019-867	#867	Tadpole	Tail clip	25	Imam Ali	32.241079, 35.169876	OR017279	OR018045
27.03.2012	GMS2019-869	#869	Tadpole	Tail clip	25	Imam Ali	32.241079, 35.169876	OR016916	OR018046
27.03.2012	GMS2019-871	#871	Tadpole	Tail clip	25	Imam Ali	32.241079, 35.169876	OR017278	OR018047
27.03.2012	GMS2019-873	#873	Tadpole	Tail clip	25	Imam Ali	32.241079, 35.169876	OR016915	OR018048
27.03.2012	GMS2019-874	#874	Tadpole	Tail clip	25	Imam Ali	32.241079, 35.169876	OR016914	OR018049
06.03.2012	GMS2019-876	#876	Tadpole	Tail clip	11	Dora	32.291801,34.847814	OR017105	OR018050
06.03.2012	GMS2019-877	#877	Tadpole	Tail clip	11	Dora	32.291801,34.847814	OR017285	OR018051
06.03.2012	GMS2019-878	#878	Tadpole	Tail clip	11	Dora	32.291801,34.847814	OR016809	OR018052
06.03.2012	GMS2019-879	#879	Tadpole	Tail clip	11	Dora	32.291801,34.847814	OR017171	OR018053
06.03.2012	GMS2019-880	#880	Tadpole	Tail clip	11	Dora	32.291801,34.847814	OR017104	OR018054
06.03.2012	GMS2019-881	#881	Tadpole	Tail clip	11	Dora	32.291801,34.847814	OR017170	OR018055
06.03.2012	GMS2019-882	#882	Tadpole	Tail clip	11	Dora	32.291801,34.847814	OR017169	OR018056
06.03.2012	GMS2019-883	#883	Tadpole	Tail clip	11	Dora	32.291801,34.847814	OR017168	OR018057
06.03.2012	GMS2019-884	#884	Tadpole	Tail clip	11	Dora	32.291801,34.847814	OR016808	OR018058
06.03.2012	GMS2019-885	#885	Tadpole	Tail clip	11	Dora	32.291801,34.847814	OR016807	OR018059
06.03.2012	GMS2019-886	#886	Tadpole	Tail clip	11	Dora	32.291801,34.847814	OR017167	OR018060
15.04.2012	GMS2019-887	#887	Tadpole	Tail clip	20	Gan Yoshya	32.345634, 35.001401	OR017166	OR018061
15.04.2012	GMS2019-889	#889	Tadpole	Tail clip	20	Gan Yoshya	32.345634, 35.001401	OR017165	OR018062
15.04.2012	GMS2019-890	#890	Tadpole	Tail clip	20	Gan Yoshya	32.345634, 35.001401	OR017164	OR018063
15.04.2012	GMS2019-891	#891	Tadpole	Tail clip	20	Gan Yoshya	32.345634, 35.001401	OR017277	OR018064
15.04.2012	GMS2019-892	#892	Tadpole	Tail clip	20	Gan Yoshya	32.345634, 35.001401	OR017163	OR018065
15.04.2012	GMS2019-893	#893	Tadpole	Tail clip	20	Gan Yoshya	32.345634, 35.001401	OR017162	OR018066
15.05.2012	GMS2019-894	#894	Tadpole	Tail clip	13	Ein Yeella	32.68506, 35.54794	OR017161	OR018067
15.05.2012	GMS2019-895	#895	Tadpole	Tail clip	13	Ein Yeella	32.68506, 35.54794	OR017276	OR018068
15.05.2012	GMS2019-896	#896	Tadpole	Tail clip	13	Ein Yeella	32.68506, 35.54794	OR017160	OR018069
15.05.2012	GMS2019-897	#897	Tadpole	Tail clip	13	Ein Yeella	32.68506, 35.54794	OR017159	OR018070

Date	Sample ID	Individual ID	Life Stage	Type of Sample	Population No.	Pool	Coordinates	GenBank accession number (16S)	GenBank accession number (COI)
15.05.2012	GMS2019-898	#898	Tadpole	Tail clip	13	Ein Yeella	32.68506, 35.54794	OR017158	OR018071
15.05.2012	GMS2019-899	#899	Tadpole	Tail clip	13	Ein Yeella	32.68506, 35.54794	OR017157	OR018072
15.05.2012	GMS2019-900	#900	Tadpole	Tail clip	13	Ein Yeella	32.68506, 35.54794	OR017156	OR018073
15.05.2012	GMS2019-901	#901	Tadpole	Tail clip	13	Ein Yeella	32.68506, 35.54794	OR017155	OR018074
15.05.2012	GMS2019-902	#902	Tadpole	Tail clip	13	Ein Yeella	32.68506, 35.54794	OR017315	OR018075
15.05.2012	GMS2019-903	#903	Tadpole	Tail clip	13	Ein Yeella	32.68506, 35.54794	OR017154	OR018076
15.05.2012	GMS2019-904	#904	Tadpole	Tail clip	13	Ein Yeella	32.68506, 35.54794	OR017153	OR018077
15.05.2012	GMS2019-905	#905	Tadpole	Tail clip	13	Ein Yeella	32.68506, 35.54794	OR017303	OR018078
15.05.2012	GMS2019-906	#906	Tadpole	Tail clip	13	Ein Yeella	32.68506, 35.54794	OR017152	OR018079
25.04.2012	GMS2019-908	#908	Adult	Toe clip	46	Yaar	32.410999, 34.898113	OR017110	OR018080
25.04.2012	GMS2019-909	#909	Adult	Toe clip	46	Yaar	32.410999, 34.898113	OR016806	OR018081
25.04.2012	GMS2019-910	#910	Adult	Toe clip	46	Yaar	32.410999, 34.898113	OR016805	OR018082
25.04.2012	GMS2019-911	#911	Adult	Toe clip	46	Yaar	32.410999, 34.898113	OR016804	OR018083
25.04.2012	GMS2019-913	#913	Adult	Toe clip	46	Yaar	32.410999, 34.898113	OR016803	OR018084
25.04.2012	GMS2019-914	#914	Adult	Toe clip	46	Yaar	32.410999, 34.898113	OR016802	OR018085
25.04.2012	GMS2019-915	#915	Adult	Toe clip	46	Yaar	32.410999, 34.898113	OR017151	OR018086
25.04.2012	GMS2019-916	#916	Adult	Toe clip	46	Yaar	32.410999, 34.898113	OR016798	OR018087
25.04.2012	GMS2019-917	#917	Adult	Toe clip	46	Yaar	32.410999, 34.898113	OR017302	OR018088
25.04.2012	GMS2019-918	#918	Adult	Toe clip	46	Yaar	32.410999, 34.898113	OR016797	OR018089
25.04.2012	GMS2019-919	#919	Adult	Toe clip	46	Yaar	32.410999, 34.898113	OR017275	OR018090
25.04.2012	GMS2019-920	#920	Adult	Toe clip	46	Yaar	32.410999, 34.898113	OR017238	OR018091
25.04.2012	GMS2019-921	#921	Adult	Toe clip	46	Yaar	32.410999, 34.898113	OR017150	OR018092
25.04.2012	GMS2019-922	#922	Adult	Toe clip	46	Yaar	32.410999, 34.898113	OR016913	OR018093
25.04.2012	GMS2019-923	#923	Adult	Toe clip	46	Yaar	32.410999, 34.898113	OR017236	OR018094
25.04.2012	GMS2019-924	#924	Adult	Toe clip	46	Yaar	32.410999, 34.898113	OR016801	OR018095
25.04.2012	GMS2019-925	#925	Adult	Toe clip	46	Yaar	32.410999, 34.898113	OR017149	OR018096
25.04.2012	GMS2019-926	#926	Adult	Toe clip	46	Yaar	32.410999, 34.898113	OR017237	OR018097

Date	Sample ID	Individual ID	Life Stage	Type of Sample	Population No.	Pool	Coordinates	GenBank accession number (16S)	GenBank accession number (COI)
25.04.2012	GMS2019-927	#927	Adult	Toe clip	46	Yaar	32.410999, 34.898113	OR017148	OR018098
05.06.2012	GMS2019-928	#928	Tadpole	Tail clip	37	Rehania	33.05097, 35.48657	OR016800	OR018099
05.06.2012	GMS2019-929	#929	Tadpole	Tail clip	37	Rehania	33.05097, 35.48657	OR017114	OR018100
05.06.2012	GMS2019-930	#930	Tadpole	Tail clip	37	Rehania	33.05097, 35.48657	OR016799	OR018101
11.04.2021	GMS2019-931	#931	Adult	Buccal swab	33	Neot Hakikar	30.934337, 35.377422	OR017322	OR018102
11.04.2021	GMS2019-932	#932	Adult	Buccal swab	33	Neot Hakikar	30.934337, 35.377422	OR017321	OR018103
11.04.2021	GMS2019-933	#933	Adult	Buccal swab	33	Neot Hakikar	30.934337, 35.377422	OR017320	OR018104
11.04.2021	GMS2019-937	#937	Adult	Buccal swab	33	Neot Hakikar	30.934337, 35.377422	OR016783	OR018105
11.04.2021	GMS2019-938	#938	Adult	Buccal swab	33	Neot Hakikar	30.934337, 35.377422	OR016784	OR018106
11.04.2021	GMS2019-940	#940	Adult	Buccal swab	33	Neot Hakikar	30.934337, 35.377422	OR016785	OR018107
11.04.2021	GMS2019-941	#941	Adult	Buccal swab	33	Neot Hakikar	30.934337, 35.377422	OR016787	OR018108
11.04.2021	GMS2019-942	#942	Adult	Buccal swab	33	Neot Hakikar	30.934337, 35.377422	OR016786	OR018109

Supplementary Table S3. Details on additional *Hyla* sequences included in the phylogenetic analyses.

Gene	Species	Country	GenBank Accession	Source
16S	*Hyla arborea*	n.a.	MN122932	Margaryan (2020)[1]
	Hyla chinensis	China	AY458593	Zhang et al. (2005)[2]
	Hyla felixarabica	**Yemen**	**GQ916789**	**Gvozdik et al. (2010)[3]**
	Hyla japonica	Japan	EF566952	Lemmon et al., (2007)[4]
	Hyla meridionalis	Spain (Canary Islands)	EF566953	Lemmon et al., (2007)[4]
	Hyla savignyi	**Syria**	**EF566954**	**Lemmon et al., (2007)[4]**
	Hyla savignyi	**Turkey**	**GQ916754**	**Gvozdik et al. (2010)[3]**
COI	*Hyla arborea*	n.a.	MN122932	Margaryan (2020)[1]
	Hyla chinensis	China	AY458593	Zhang et al. (2005)[2]
	Hyla felixarabica	**Yemen**	**GQ916789**	**Gvozdik et al. (2010)[3]**
	Hyla japonica	Japan	HM439207	Jang et al. (2011)[5]
	Hyla meridionalis	n.a.	MK511412	Ehl et al. (2019)[6]
	Hyla savignyi	**Turkey**	**MK491947**	**Kalayci, T., 2019[1]**

[1] Unpublished

[2] Zhang,P., Zhou,H., Chen,Y.Q., Liu,Y.F. and Qu,L.H. Mitogenomic perspectives on the origin and phylogeny of living amphibians. *Syst. Biol.* **54**, 391-400 (2005)

[3] Gvozdik,V., Moravec,J., Klutsch,C. and Kotlik,P. Phylogeography of the Middle Eastern tree frogs (Hyla, Hylidae, Amphibia) as inferred from nuclear and mitochondrial DNA variation with a description of a new species. *Mol. Phylogenet. Evol.* 55, 1146-1166 (2010)

[4] Lemmon, E. M., Lemmon, A. R., Cannatella, D. C. Geological and climatic forces driving speciation in the continentally distributed trilling chorus frogs (*Pseudacris*). *Evolution: International Journal of Organic Evolution* **61**, 2086–2103 (2007).

[5] Jang,Y., Hahm,E.H., Lee,H.J., Park,S., Won,Y.J. and Choe,J.C. Geographic variation in advertisement calls in a tree frog species: gene flow and selection hypotheses. *PLoS ONE* 6, E23297 (2011)

[6] Ehl,S., Vences,M. and Veith,M. Reconstructing evolution at the community level: A case study on Mediterranean amphibians. *Mol. Phylogenet. Evol.* 134, 211-225 (2019)
